# Deubiquitylase OTUD3 prevents Parkinson’s disease through stabilizing iron regulatory protein 2

**DOI:** 10.1038/s41419-022-04704-0

**Published:** 2022-04-30

**Authors:** Fengju Jia, Hongchang Li, Qian Jiao, Chaonan Li, Lin Fu, Chunping Cui, Hong Jiang, Lingqiang Zhang

**Affiliations:** 1 Department of Physiology, Shandong Provincial Key Laboratory of Pathogenesis and Prevention of Neurological Disorders and State Key Disciplines, Physiology, Medical College of Qingdao University, Qingdao, 266071 China; 2 State Key Laboratory of Proteomics, National Center of Protein Sciences (Beijing), Beijing Institute of Lifeomics, Beijing, 100850 China

**Keywords:** Ubiquitylation, Parkinson's disease

## Abstract

Iron deposits are neuropathological hallmark of Parkinson’s disease (PD). Iron regulatory protein 2 (IRP2) is a key factor in regulating brain iron homeostasis. Although two ubiquitin ligases that promote IRP2 degradation have been identified, the deubiquitylase for stabilization of IRP2 in PD remains undefined. Here, we report OTUD3 (OTU domain-containing protein 3) functions as a deubiquitylase for IRP2, interacts with IRP2 in the cytoplasm, de-polyubiquitylates, and stabilizes IRP2 protein in an iron-independent manner. Depletion of OTUD3 results in a disorder of iron metabolism. OTUD3 knockout mice display nigral iron accumulation, motor deficits, and nigrostriatal dopaminergic neurodegeneration, which resembles the pathology of PD. Consistently, decreased levels of OTUD3 are detected in transgenic PD mice expressing A53T mutant of human α-synuclein. Five single nucleotide polymorphism mutations of OTUD3 are present in cases of sporadic PD or controls, although no significant associations of OTUD3 SNPs with sporadic PD are detected. Taken together, these findings demonstrate that OTUD3 is a bona fide deubiquitylase for IRP2 and plays a critical role in the nigral iron deposits in PD.

## Introduction

Parkinson’s disease (PD) is one of the most common movement disorders characterized by decreased of dopaminergic neurons. Various factors have been considered as contributing to the degeneration of dopaminergic neurons in PD, such as oxidative stress, mitochondria dysfunction, iron deposition, inflammation, and aberrant protein aggregation [1–6]. Among these, elevated iron is attracting increasing attention [7]. More noticeably, both neuropathological and imaging studies have demonstrated iron selectively accumulates in the substantia nigra (SN) of patients or animal model with PD [8–14].

Iron is an essential nutrient element required in cellular biosynthetic and metabolic processes [15]. Intracellular iron levels are elaborately balanced by iron efflux, uptake, and storage proteins that are regulated by iron regulatory proteins (IRPs, including IRP1 and IRP2) [16]. Under various iron conditions, IRPs could bind to IREs in the targeted mRNA, stabilize the mRNA or prevent its translation. Although IRP1 and IRP2 share high sequence homology, IRP1 is mainly expressed in kidney, liver, and brown fat, whereas IRP2 mainly in the central nervous system [17]. Furthermore, most of intrinsic IRP1 functions as a cytosolic aconitase that lacks IRE binding activity rather than an RNA-binding protein in vivo [17]. Whereas IRP2 is sensitive to iron status and can compensate for the loss of IRP1 by increasing its binding activity under normal circumstances [17]. Simultaneous knockout of both IRP1 and IRP2 leads to embryonic lethality in mice. Single knockout studies showed that *IRP2*^−/−^ mice develop a neurodegenerative phenotype associated with mis-regulation of iron metabolism in a specific area of the brain [18–20], whereas *IRP1*^−/−^ mice are spared [17, 21, 22]. Importantly, *IRP2*^−/−^ mice display iron metabolism disorders throughout the brain and duodenal mucosa, resulting in increased expression of ferritin and ferroportin and increased iron content [18, 19], indicating the critical role of IRP2 in the maintenance of iron homeostasis. Recently, two patients with mutations in IREB2 have been identified to be exhibiting early-onset and progressive neurological disease. And the patients’ cellular abnormalities were reversed by lentiviral-mediated restoration of IRP2 expression [23, 24]. We previously showed that decreased IRP2 levels were observed in 1-methyl-4-phenylpyridinium (MPP^+^)-treated dopaminergic cells [25], supporting the physiological role of IRP2 in the control of iron balance. Intracellular IRP2 is mainly degraded through the ubiquitin-proteasome pathway. Two E3 ubiquitin ligases that promote IRP2 polyubiquitylation for degradation have been identified as F box and leucine-rich repeat protein 5 (FBXL5) [26, 27] and heme-oxidized IRP2 ubiquitin ligase 1 (HOIL-1) [28, 29]. However, the stabilization mechanism and the deubiquitylase (DUB) for IRP2 remain unclear yet.

There are about 100 deubiquitylating enzymes (DUBs) found in the human proteome, which consists of six families: USPs, OTUs, JAMMs, MJDs, MINDYs, and UCHs. Recently, we demonstrated the OTU (ovarian tumor protease) family member OTUD3 (OTU domain-containing protein 3) suppresses the development of breast cancer via maintaining the stability of PTEN [30], but promotes the development of lung cancer through stabilizing GRP78 [31]. Additional evidence suggested OTUD3’s conclusive associations with ulcerative colitis [32], the ribosome-associated quality control pathway [33], and innate antiviral immune signaling [34]. The DUB activity of OTUD3 is inhibited by some virus [34]. It is possible that a viral infection is a trigger or contributor to PD [35], therefore, the ubiquitination or deubiquitation may play a role in the occurrence and development of PD. Current studies show that OTUD3 acts as a potent DUB in multiple cancer pathogenesis, however, the comprehensive understanding of the role of OTUD3 in neurodegenerative disorders is still limited. In the present study, OTUD3 is verified to be involved in the dysregulation of iron metabolism in PD. OTUD3 is the potent DUB for iron regulatory protein 2 (IRP2), implying the roles of OTUD3 in neurodegenerative disorders beyond multiple cancer pathogenesis.

## Results

### *OTUD3*^−/−^ mice displayed impaired motor coordination and neuronal degeneration in the nigrostriatal system

**Fig. 1:**
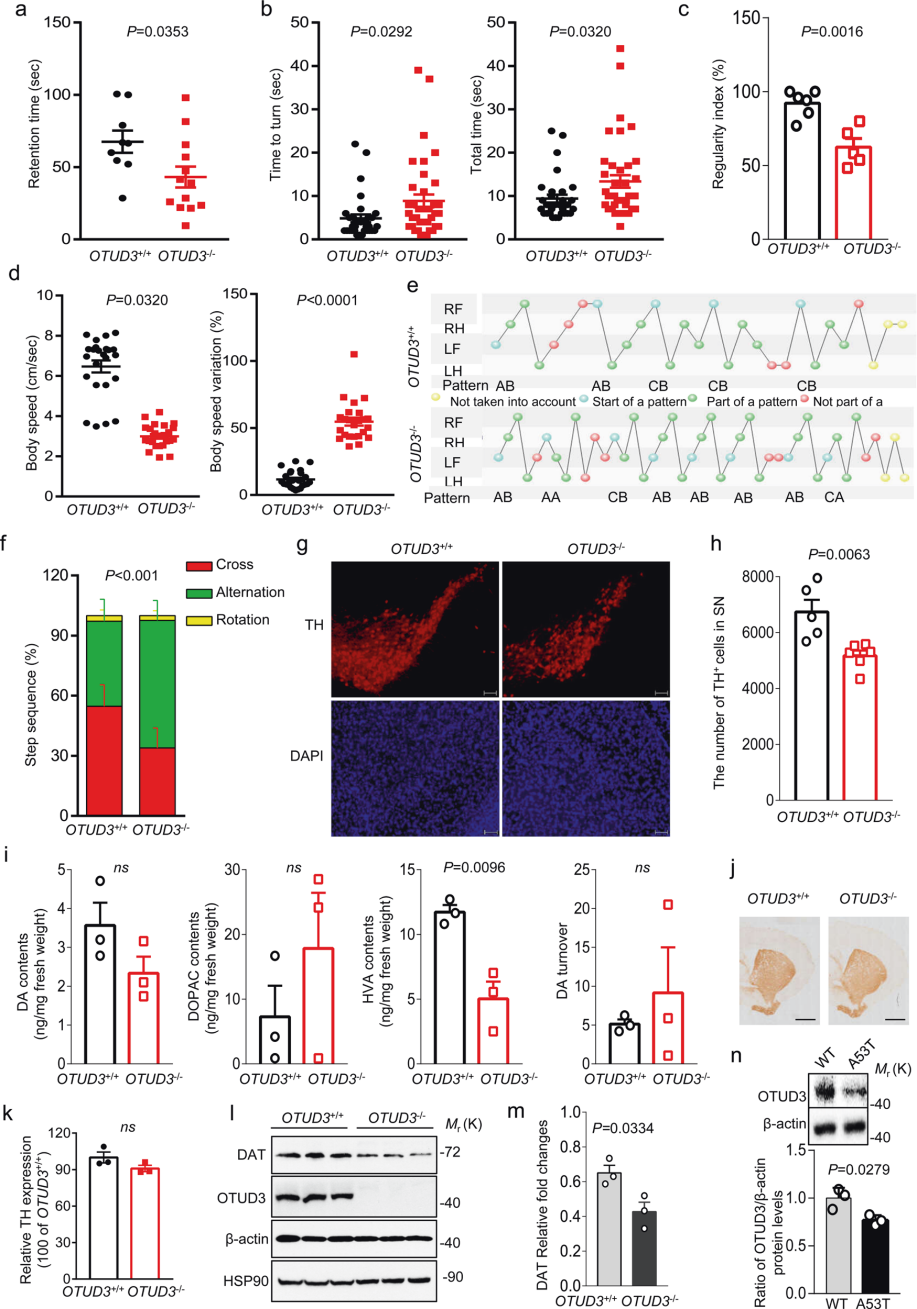
*OTUD3*^−/−^ mice displayed impaired motor coordination and neuronal degeneration in the nigrostriatal system. **a** The residence time of mice (*OTUD3*^+/+^: *n* = 9; *OTUD3*^−/−^: *n* = 13) on rotarod treadmills was assessed by the rotarod test. **b** The time to turn to orient downwards (*OTUD3*^+/+^: *n* = 32; *OTUD3*^−/−^: *n* = 37) and total time in the pole test (*OTUD3*^+/+^: *n* = 32; *OTUD3*^−/−^: *n* = 39) were evaluated. **c**–**f** Measurement of gait analysis was conducted using a Catwalk^TM^ gait analysis system (number of mice: *OTUD3*^+/+^: *n* = 6; *OTUD3*^−/−^: *n* = 5 in (**c**); *OTUD3*^+/+^: *n* = 24; *OTUD3*^−/−^: *n* = 24 in (**d**); *OTUD3*^+/+^: *n* = 6; *OTUD3*^−/−^: *n* = 6 in (**f**). LF, left front; RF, right front; LH, left hind; RH, right hind. **g** Representative imaging of TH-positive neurons in the SN using immunochemistry assay. Scale bars, 100 μm. **h** Show a quantification of TH-positive neurons in the SN of indicated mice. *OTUD3*^+/+^: *n* = 5; *OTUD3*^−/−^: *n* = 6. **i** DA contents and its metabolites in the striatum were determined by HPLC-ECD. *OTUD3*^+/+^: *n* = 3; *OTUD3*^−/−^: *n* = 3. **j** The TH-immunoreactive fibers in the striatum of mice. *OTUD3*^+/+^: *n* = 3; *OTUD3*^−/−^: *n* = 3. Scale bars, 1 mm. **k** The quantification of TH-immunoreactive fibers in the striatum by ImageJ. *n* = 3; *OTUD3*^−/−^: *n* = 3. **l** DAT protein levels were assessed in the SN of mice. *OTUD3*^+/+^: *n* = 3; *OTUD3*^−/−^: *n* = 3. **m** The quantification of DAT protein levels of SN of mice. *OTUD3*^+/+^: *n* = 3; *OTUD3*^−/−^: *n* = 3. **n** OTUD3 protein levels were assessed in the SN of A53T transgenic mice. WT: *n* = 3; A53T: *n* = 3. Data are depicted as bar graphs with mean ± s.e.m. Student’s *t*-test (**a**–**d, h**, **i, m**, **n**); one-way ANOVA test (**f**). The statistics data and uncropped western blots can be found in Supplemental Material.

*OTUD3*^−/−^ mice are originally bred to focus on the role of OTUD3 in tumorigenesis of breast cancer and lung cancer. It was a delight to find that motor deficits are indicative of the problems in coordinated movement in *OTUD3*^−/−^ mice at the age of 24 months. In detail, compared with wild type (*OTUD3*^+/+^) mice, the residence time of *OTUD3*^−/−^ mice at 24 months on rotarod treadmills is shortened (Fig. 1a), whereas the time to turn to orient downwards and total time in the pole test were prolonged (Fig. 1b). Moreover, the step sequence regularity index (a fractional measure of inter-paw coordination) was significantly decreased (Fig. 1c). Reduced velocity of movement was also observed in *OTUD3*^−/−^ mice at 24 months, although the speed variation showed a sharp elevation (Fig. 1d). In gait, the main step sequence of *OTUD3*^−/−^ mice was changed from cross pattern to alternation pattern (Fig. 1e, f). Moreover, Dynamic (Supplementary Fig. 1a) and static paw parameters (Supplementary Fig. 1b, c) are also impaired in *OTUD3*^−/−^ mice at 24 months of age.

Considering PD is attended by a constellation of disabling motoric deficits accompanied by progressive degeneration of substantia nigra and depletion of dopamine in the striatum, we further evaluated the state of nigrostriatal system of *OTUD3*^−/−^ mice. Strikingly, compared with WT mice, there is a 23.3% loss of the dopaminergic neurons in the SN of *OTUD3*^−/−^ mice (Fig. 1g, h). A 57.1% decrease of HVA contents in the striatum was also observed (Fig. 1i). No difference was observed in the striatal DA contents in *OTUD3*^−/−^ mice at 24 months, although there is a downward tendency (Fig. 1i). Although the decline of projecting fibers of dopaminergic neurons in striatum was not detected in aged *OTUD3*^−/−^ mice (Fig. 1j, k), Compared with the corresponding *OTUD3*^+/+^ mice, the protein levels of DAT decrease (Fig. 1l, m).

Transgenic mice expressing mutant A53T of human α-synuclein is a common animal model used for PD research. These A53T transgenic mice showed delayed residence time on rotarod treadmills (Supplementary Fig. 1d) and longer time to turn to orient downwards at the age of at 6 months (Supplementary Fig. 1e). Six-month-old A53T transgenic mice also showed degeneration of nigrostriatal dopaminergic neurons, which was indicated by the decreased levels of tyrosine hydroxylase (TH) in the SN (Supplementary Fig. 1f) and striatal DA contents (Supplementary Fig. 1g). Meanwhile, the decreased protein level of OTUD3 was observed in the SN of these mice (Fig. 1n).

Considering ventral tegmental area (VTA) is the other one of two main dopamine neural pathway, we detect the number of DA neurons in VTA. No changes were examined in the number of TH^+^ neurons in VTA (Supplementary Fig. 2a, b). We also observed the variations of gamma-amino butyric acid (GABA)ergic neurons in striatum and cholinergic neurons in medial septal area (MS), respectively. No significant differences were detected in the number of the striatal glutamic acid decarboxylase-67 (GAD67) positive neurons (Supplementary Fig. 2c, d) and choline acetyl transferase (ChAT) positive neurons of MS (Supplementary Fig. 2e, f). The results indicated that the neuron degeneration is specific for DAergic neurons of the SN and does not affect other neuronal types.

The age-dependent vulnerability of dopaminergic neurons is an important hallmark of PD. We observed the quantification of TH^+^ neurons cell bodies in the SN and their projecting fibers in striatum in young (1.5 months) and adult (3 months). Compared with the corresponding *OTUD3*^+/+^ mice, no changes were observed in the number of TH^+^ neurons in the SN (Supplementary Fig. 3a–d) and TH intensity in the striatum (Supplementary Fig. 3e, h) in young and adult *OTUD3*^−/−^ mice, which implying that the loss of dopaminergic neurons inducing by deletion of OTUD3 is indeed age-dependent. As a matter of fact, the above-mentioned lesions of nigrostriatal system and motor phenotype of *OTUD3*^−^^/−^ mice at 24 months resembled the symptoms of PD.

### OTUD3 deletion induced disorder of iron metabolism and reduced IRP2 protein levels

**Fig. 2:**
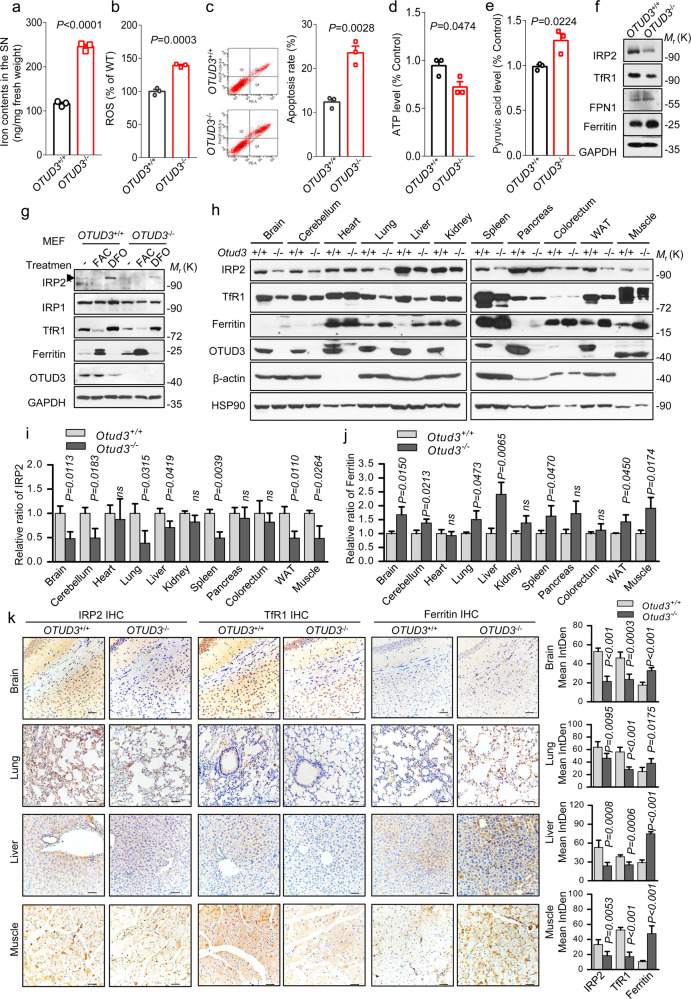
OTUD3 deletion induced disorder of iron metabolism. **a** Iron contents were detected in the SN of mice at 24 months. *OTUD3*^+/+^: *n* = 3; *OTUD3*^−/−^: *n* = 3. **b**, **c** Reactive oxygen species (ROS) and cell apoptosis rate were measured in MEFs. ATP (**d**) and pyruvic acid (**e**) levels of SN from mice at 24 months. SN tissues from two mice were mixed as one sample, *OTUD3*^+/+^: *n* = 3; *OTUD3*^−/−^: *n* = 3. **f** Changes of iron transport-related proteins in the SN of mice at 24 months. *OTUD3*^+/+^: *n* = 3; *OTUD3*^−/−^: *n* = 3. **g** The effects of 100 µg/ml FAC or 100 µg/ml DFO on iron transport-related proteins in MEFs. **h** Changes of iron transport-related proteins in various organs of indicated mice. **i** The quantification of IRP2 protein levels in various organs of indicated mice. *OTUD3*^+/+^: *n* = 3; *OTUD3*^−/−^: *n* = 3. **j** The quantification of ferritin protein levels in various organs of indicated mice. *OTUD3*^+/+^: *n* = 3; *OTUD3*^−/−^: *n* = 3. **k** The expression of iron transport-related proteins in various organs of *OTUD3*^+/+^ and *OTUD3*^−/−^ mice by immunohistochemistry assay. Scale bars, 50 μm. All panels are representative results of three or more independent experiments. Data shown are mean ± s.e.m. Student’s *t*-test. The statistics data and uncropped western blots can be found in Supplemental Material.

α-synuclein plays a key role in the process of neurodegeneration, however, we did not detect obviously elevated levels of α-synuclein and phosphorylated α-synuclein in SN of *OTUD3*^−/−^ mice (Supplementary Fig. 4a–e). α-synuclein protein levels remained unchanged in neurons with overexpression of OTUD3 (Supplementary Fig. 4f). Although microglia-mediated neuroinflammation is one of the most striking hallmarks of PD, no obvious activation of microglia in SN of *OTUD3*^−/−^ mice compared with the corresponding *OTUD3*^+/+^ mice (Supplementary Fig. 4g). Considering that iron accumulates specifically in SN in the early stage of PD, we explored the influence of iron metabolism disorders caused by OTUD3 on neurons. Delightedly, iron contents are increased approximately by two folds in the SN of *OTUD3*^−/−^ mice at 24 months (Fig. 2a), whereas no iron deposits were observed in the cortex or hippocampus (Supplementary Fig. 5a). There are no significant changes in the copper contents in the SN, cortex, or hippocampus (Supplementary Fig. 5b). Iron content is increased in the *OTUD3*^−/−^ embryonic fibroblasts (MEFs) (Supplementary Fig. 5c), this phenomenon was also observed in the lung and the stomach among peripheral tissues (Supplementary Fig. 5d). We found that deletion of OTUD3 resulted in increased intracellular reactive oxygen species (ROS), and decreased mitochondrial transmembrane potential (ΔΨm) (Fig. 2b and Supplementary Fig. 5e). Elevated apoptosis rate was also detected (Fig. 2c). Elevated apoptosis rate is also detected (Fig. 2c). The levels of superoxide dismutase 1 (SOD1) and Bcl-2 were observed to be declining (Supplementary Fig. 5h), although no significant changes were observed in levels of p53 in SN of *OTUD3*^−/−^ mice (Supplementary Fig. 5f, g). While intracellular ATP and energetic metabolites pyruvate levels are decreased in *OTUD3*^−/−^ mice relative to the *OTUD3*^+/+^ mice (Fig. 2d, e).

Generally, cellular iron uptake mainly occurs through transferrin receptor 1 (TfR1) and divalent metal transporter 1 (DMT1). Ferroportin 1 (FPN1) is the only known iron transporter responsible for intracellular iron export, and ferritin provides a form of iron storage. These proteins mentioned above are regulated by IRP2 through binding to IREs in their respective messenger RNAs (mRNAs), which dominates brain iron homeostasis. Although increased levels of DMT1 have been directly correlated to PD pathology in many PD animal models, we did not observe the up-regulation of DMT1 in SN of *OTUD3*^−/−^ mice as compared with *OTUD3*^+/+^ mice (Supplementary Fig. 5i, j). In 24-month-old *OTUD3*^−/−^ mice, decreased levels of IRP2 were detected in the SN (Fig. 2f) and MEF cells (Supplementary Fig. 5k), accompanied by increased levels of ferritin. Furthermore, the changes of protein levels of IRP2, TfR1 and ferritin caused by OTUD3 are regulated by ferric ammonium citrate (FAC) and deferoxamine (DFO) (Fig. 2g). With overexpression of OTUD3, we observed increased IRP2 and TfR1, and decreased ferritin in neuroblastoma cell line SH-SY-5Y. In contrast, decreased IRP2 and TfR1, and increased ferritin were observed in SH-SY-5Y cells with siOTUD3 (Supplementary Fig. 5l). These changes in levels of IRP2, TfR1, and ferritin were also observed in various organs of *OTUD3*^−/−^ mice (Fig. 2h–j). As expected, decreased expressions of IRP2 and TfR1, and increased expression of ferritin were also detected in organs of *OTUD3*^−^^/−^ mice by immunohistochemistry assay (Fig. 2k and Supplementary Fig. 5m).

### OTUD3 was the DUB that maintained IRP2 stability

**Fig. 3:**
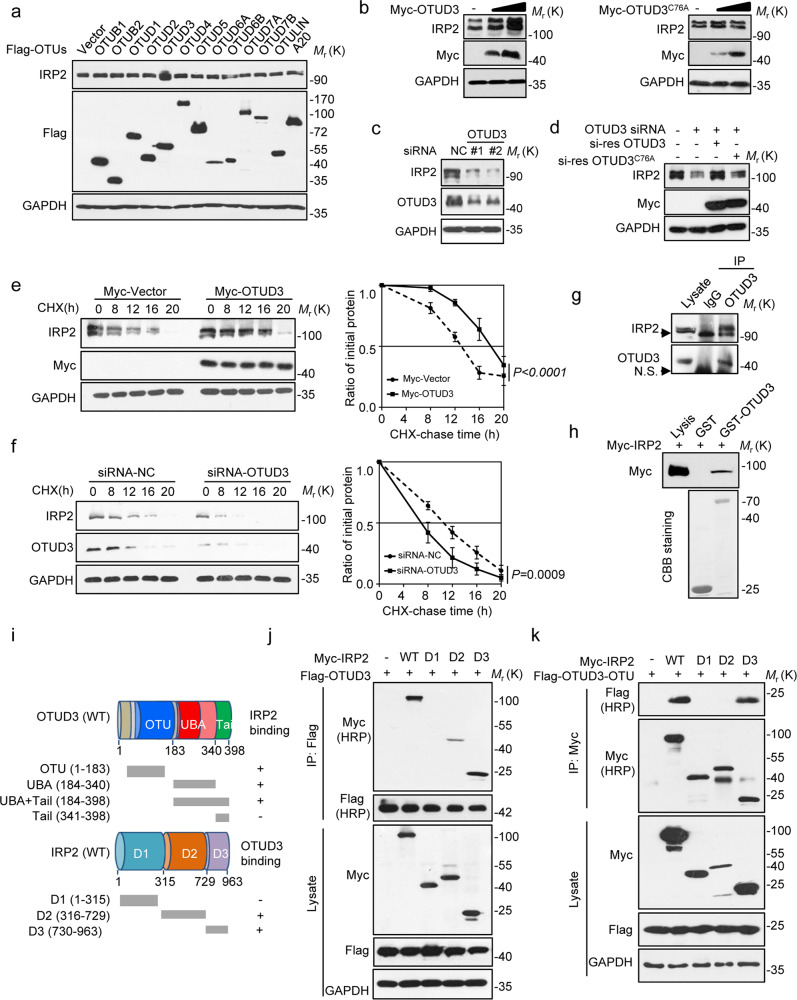
OTUD3 was the DUB that maintained IRP2 stability. **a** The indicated OTU subfamily of DUBs was each transfected into HEK293T cells, and then the protein level of IRP2 were detected. **b** Increasing amount of OTUD3 WT or C76A were transfected into HEK293T cells and IRP2 expression was detected. **c** OTUD3 was depleted by siRNA in HEK293T cells, IRP2 levels were analyzed. **d** siRNA-resistant (si-res) OTUD3 WT or C76A was introduced into HEK293T cells together with OTUD3 siRNA. IRP2 levels were measured. **e**, **f** HEK293T cells transfected with the indicated plasmids or siRNA were treated with cycloheximide (10 µg/ml). Quantification of IRP2 levels relative to GAPDH was shown. Results were shown as mean ± s.e.m. *n* = 3 independent experiments, two-way ANOVA test. **g** HEK293T cell lysates were subject to immunoprecipitation with control IgG or anti-OTUD3 antibodies. The immunoprecipitates were then blotted. **h** The interaction of OTUD3 and IRP2 was assessed by GST pull-down assay. **i** Overview of OTUD3 and IRP2 structures. **j** Co-IP assays were performed to map the domain of IRP2 required for interaction with OTUD3. **k** Co-IP assays were performed to map the domain of IRP2 required for interaction with OTUD3-OTU. All panels are representative results of three or more independent experiments. The statistics data and uncropped western blots can be found in Supplemental Material.

Since IRP2 coordinates the intracellular iron balance by regulating the expressions of the above proteins, we performed a whole DUB library screen (USPs, OTUs, UCHs, MJDs, JAMMs, and MCPIPs) in HEK293T cells and identified that OTU domain-containing protein 3 (OTUD3) remarkably upregulated IRP2 levels, while other DUBs did not (Fig. 3a and Supplementary Fig. 6a). OTUD3 regulates IRP2 levels in a dose-dependent manner, requiring its DUB enzyme activity because the catalytically inactive mutant OTUD3-C76A lost this ability (Fig. 3b). Depletion of endogenous OTUD3 markedly decreased IRP2 levels (Fig. 3c). Overexpression of an siRNA-resistant WT OTUD3, rather than the C76A mutant, reversed the decrease of IRP2 levels induced by OTUD3 knockdown (Fig. 3d). Treatment with the proteasome inhibitor MG132 also reversed the decrease of IRP2 (Supplementary Fig. 6b). OTUD3 overexpression or depletion had no significant effect on IRP2 mRNA levels (Supplementary Fig. 6c, d). IRP2 stability affected by OTUD3 was assessed by the protein synthesis inhibitor cycloheximide (CHX). The half-life of IRP2 was prolonged in cells that overexpressed OTUD3 (Fig. 3e) but shortened in cells that were depleted of OTUD3 (Fig. 3f).

Subsequently, the interaction between OTUD3 and IRP2 was determined. Endogenous co-immunoprecipitation (Co-IP) assay, GST pull-down assay, and exogenous Co-IP assay all showed that OTUD3 is able to interact with IRP2 (Figs. 3g, 1h and Supplementary Figs. 6e, 1f). Immunofluorescence analysis showed that endogenous OTUD3 and IRP2 are colocalized in the cytosol of HEK293T cells (Supplementary Fig. 6g). Both the OTU domain and the UBA (ubiquitin associated) region of OTUD3 are sufficient to interact with IRP2 (Fig. 3j). Additionally, we mapped the OTUD3-binding region of IRP2 are the D2 and D3 regions (Fig. 3k).

### OTUD3 catalyzed the deubiquitylation of IRP2

**Fig. 4:**
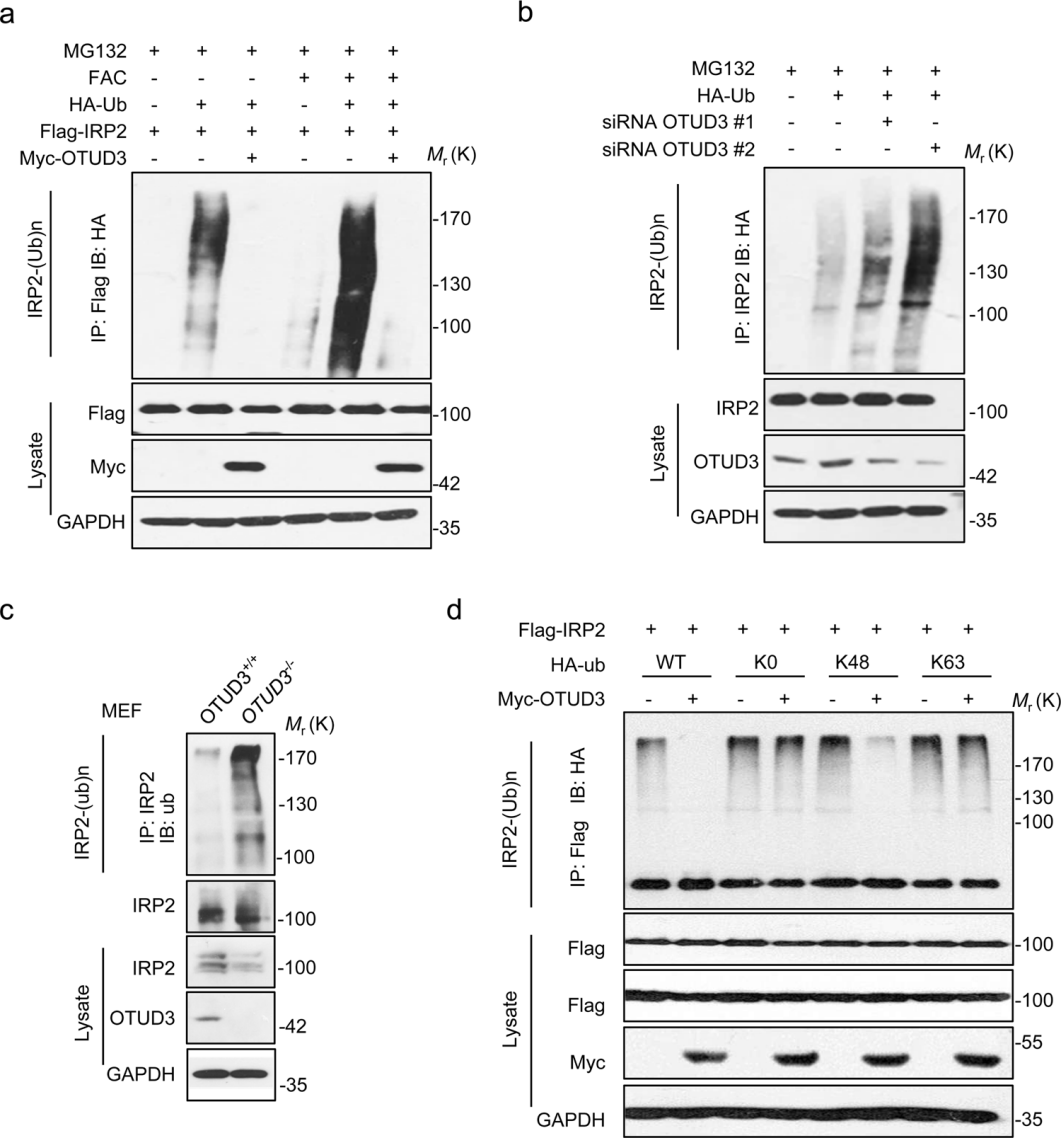
OTUD3 de-polyubiquitylated IRP2. **a**, **b** HEK293T cells transfected with the indicated constructs or siRNA were treated with MG132 for 8 h before collection with 100 µg/ml FAC or not. **c** IRP2 ubiquitylation was analyzed in *OTUD3*^−/−^ MEFs. **d** The IRP2 ubiquitylation linkage was analyzed in HEK293T cells transfected with IRP2, OTUD3, and the indicated ubiquitin Lys 0, Lys 48-only, or Lys 63-onlyplasmids. All panels are representative results of three or more independent experiments. The uncropped western blots can be found in Supplemental Material.

Next, we examined whether OTUD3 catalyzes the deubiquitylation of IRP2. Indeed, ubiquitylation assays showed that ectopic expression of OTUD3 efficiently removes IRP2 ubiquitin chains (Fig. 4a). Conversely, downregulation of endogenous OTUD3 results in increased the IRP2 ubiquitylation (Fig. 4b). Compared with the WT mice, *OTUD3*^−/−^ MEFs showed elevated ubiquitylation of IRP2 (Fig. 4c). Overexpression of OTUD3 decreased IRP2 ubiquitylation induced by its E3 ligase HOIL-1 or FBXL5, although FBXL5 could induce stronger ubiquitylation of IRP2 than HOIL-1 (Supplementary Fig. 7a, b). The level of IRP2 regulated by HOIL-1 is affected by iron concentration (Supplementary Fig. 7c), whereas less influence was observed in IRP1 (Supplementary Fig. 7d). The main function of K48-type ubiquitin chain is to mediate proteasome degradation, while K63-type ubiquitin chain mainly regulates protein activity, transport, localization, and cell endocytosis. Most OTUs show intrinsic linkage specificity, preferring one or a small defined subset of Ub linkage types. OTUD3 effectively removes K48-linkage poly-chains but not monoubiquitylation and the non-degradative K63-linkage poly-chains of IRP2 (Fig. 4d).

### OTUD3 stabilized IRP2 in an iron-independent manner

**Fig. 5:**
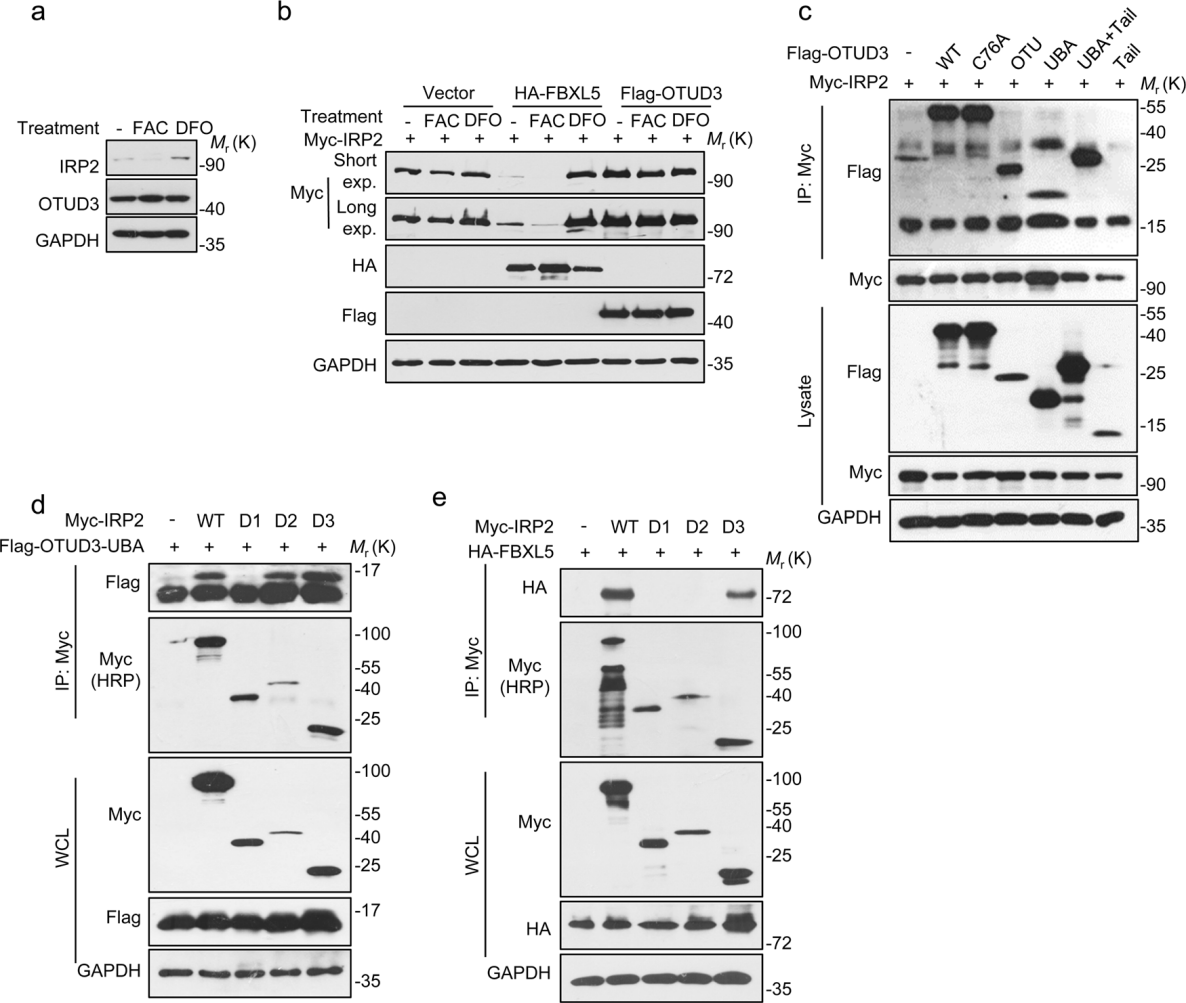
OTUD3 stabilized IRP2 in an iron-independent manner. **a** The expression of OTUD3 and IRP2 regulated by 100 µg/ml FAC or 100 µg/ml DFO treatment. **b** The expression of IRP2 with FBXL5 or OTUD3 in various iron status. **c**, **d** Co-IP assays were performed to map the interactive domains with IRP2 and OTUD3. **e** Co-IP assays were performed to map the interactive domains with IRP2 and FBXL5. All panels are representative results of three or more independent experiments. The ucropped western blots can be found in Supplemental Material.

Interestingly, the stability of IRP2 itself is regulated by iron, which is accumulated under iron depleted condition and degraded upon iron repletion (Fig. 5a). However, OTUD3 levels were not affected by the treatment of FAC or DFO (Fig. 5a). Current evidences suggest that FBXL5, an F-box ubiquitin ligase, ubiquitylates IRP2 in an iron-dependent manner, while FBXL5 itself is stabilized by iron at an elevated level. IRP2 was constantly stabilized by overexpressed OTUD3 despite various iron status (Fig. 5b). FBXL5 has a hemerythrin-like domain in its N terminus that directly binds to iron, however, no domains responsive to iron are observed in OTU of OTUD3 (Supplementary Fig. 8). CO-IP showed that the D3 region of IRP2 interacted with the OTU domain of OTUD3 (Fig. 5c), while D2 and D3 of IRP2 interacted with the UBA domain of OTUD3 (Fig. 5d). Considering IRP2-D3 is the binding region of FBXL5 as well (Fig. 5e), a competition might reside between OTUD3 and FBXL5.

### No significant mutations were detected in OTUD3 SNPs in sporadic PD patients

**Fig. 6:**
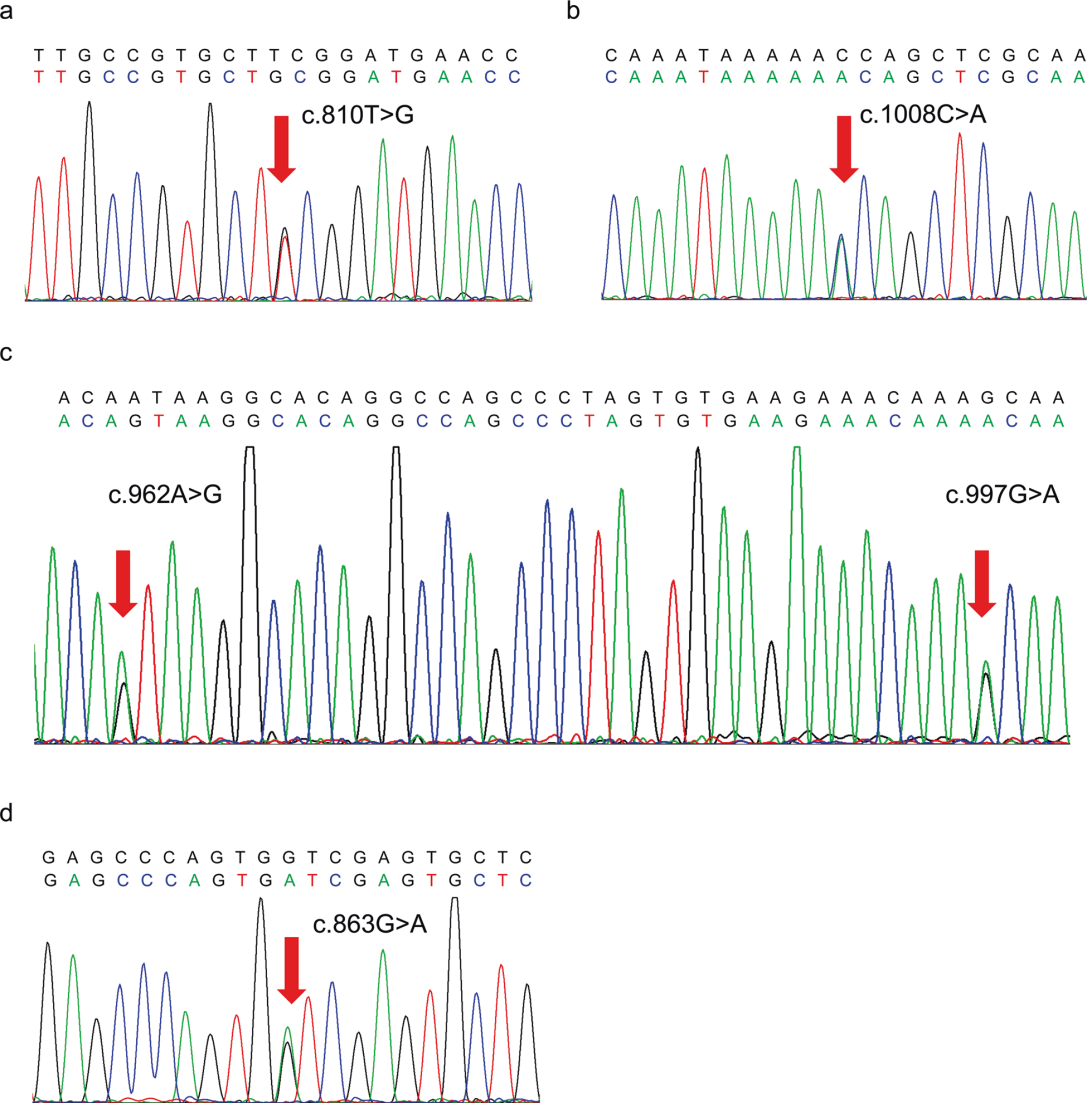
Confirmation of inconsistent genotypes detected using Sanger sequencing. **a** A substitution of T with G was confirmed in the OTUD3 gene in sample PD62. **b** A heterozygous substitution of C with A was confirmed in the OTUD3 gene in sample PD8. **c** Substitution of A with G and substitution of G with A were confirmed in the OTUD3 gene in sample Control41. **d** A substitution of G with A was confirmed in the OTUD3 gene in sample Control65.

Owing to its potential role in PD, OTUD3 mutations of the whole exons were further investigated in blood samples of cases of sporadic PD and the controls by Sanger sequencing. The sequencing data showed that both the PD patients and the controls harbor five single nucleotide polymorphism mutations of OTUD3 (rs75742716 T > G, c.1008C > A, rs2298110 A > G, rs10916668 A > G and rs78466831 A > G) (Fig. 6a–d), indicating that OTUD3 N321S, A333T, and G288D mutant proteins might be expressed. The first two missense mutations are detected alone in cases of PD, whereas the remaining three mutated SNPs are identified in both PD patients and the controls (Supplementary Table 1). SNPs rs75742716 T > G (alternation frequency: 0.003) and rs78466831 A > G (alternation frequency: 0.004) are rare mutations in Chinese population by mapping to the China Metabolic Analytics Project (ChinaMAP), whereas c.1008C > A is not recorded. Although we explored the whole exon sequencing of OTUD3, no significant associations of OTUD3 SNPs with sporadic PD were observed (Supplementary Table 2).

## Discussion

Gross dyskinesia is a key diagnostic character of PD patients which represents prominent and disabling aspects of the disorder [36]. Amazingly, motor deficits are indicative of problems in coordinated movement in *OTUD3*^−/−^ mice compared to WT mice. In *OTUD3*^−^^/−^ mice, we noted a specific loss of the dopaminergic neurons in the SN, and other neuronal types were not involved. Furthermore, the loss of dopaminergic neurons inducing by deletion of OTUD3 is indeed age-dependent. Furthermore, this is the first report to show that *OTUD3*^−/−^ mice exhibits motor and non-motor PD symptoms, which provides a phenotype for *OTUD3*^−/−^ mice.

The activity and stability of intracellular IRP2 are exquisitely regulated by ubiquitinated modifications. Studies have demonstrated that the E3 ubiquitin ligase F box and leucine-rich repeat protein 5 (FBXL5) both promote the ubiquitylation and degradation of IRP2, as well as maintaining the function of hematopoietic stem cells [37] and proliferation of neural stem progenitor cells [38]. Mechanistically, OTUD3 is involved in deubiquitylating and stabilizing IRP2. These results revealed IRP2 as a substrate of OTUD3 deubiquitylase and expanded the physiological function of OTUD3 beyond tumor-related mechanisms.

Neuropathological and imaging studies also demonstrated selective iron deposits in the SN of PD patients, as well as animal models with PD [11, 12]. Currently, the mechanism in which iron accumulates specifically in SN in the early stage of PD is still unclear. It was a delight to find that iron contents were increased in the SN of *OTUD3*^−/−^ mice, which is consistent with the brain autopsy report of PD. As the cause of iron accumulation in neurons is yet to be discovered, our study might shed light on the potential mechanisms involved in the nigral iron deposits and PD. OTUD3 regulates iron metabolism by maintaining IRP2 protein stability, a key molecule of brain iron homeostasis, in an iron-independent manner. In the current study, we found out that IRP2 is a novel substrate of OTUD3, providing a new perspective for the understanding of OTUD3.

In addition to intracellular iron concentration, there are a variety of factors affecting the expression of IRP2, such as hypoxia, oxidative stress, inflammatory, ascorbic acid, and nitric oxide [39]. Ripa R et al. unveiled that microRNA-29 could regulate IRP2 expression directly and its antagonism could abrogate age-dependent repression of IRP2 in neurons in turquoise killifish [40]. IRP2 was ubiquitinated by FBXL5 and HOIL-1 [26, 41]. *FBXL5*^−/−^ mice failed to sense intracellular iron levels and died in utero at embryonic day 8.5 as a result of iron overload and subsequent oxidative stress [38], whereas *HOIL-1*^−/−^ mice exhibited amylopectin-like deposits in the myocardium, pathogen-specific immunodeficiency [42]. We identified OTUD3 as the first deubiquitylase for IRP2, and *OTUD3*^−/−^ mice resembled the motor deficits of PD.

Up to now, the variety of OTUD3 in PD has not been discovered (www.pdgene.com). Here, we present the first full exome analysis of OTUD3 of PD. The resulting reads were mapped to the human genome reference hg19. Potential pathogenic mutations were subsequently confirmed by Sanger sequencing. 5 candidate SNP mutations for OTUD3 from blood samples of sporadic PD patients and controls in a Chinese population were identified. Previously, we found that human breast cancer harbored an E86K mutation and an R79T/E86K double mutation. However, neither R79T nor E86K was detected in PD patients in our study. Tissue specificity, small sample, and posttranslational modifications of OTUD3 are possible explanations for the negative results in our study. It is still challenging to predict the functional relevance of a specific genetic mutation to PD without the appropriate functional studies or the expressions of OTUD3 in clinical patients SN.

## Materials and methods

### Ethics approval and consent to participate

This study was carried out in strict accordance with the recommendations in the “Guide for the Care and Use of Laboratory Animals of the National Institutes of Health” and the “Principles for the Utilization and Care of Vertebrate Animals”. All animal work was approved by the Institutional Animal Care and Use Committee (IACUC) at the Beijing Institute of Lifeomics. All efforts were made to minimize animal suffering. Informed consent was obtained from all individual participants included in the study. The protocol of this study was carried out in accordance with the Helsinki Declaration and approved by the Ethical Committee of the Medical College of Qingdao University.

### Plasmids and antibodies

The human deubiquitylase library was purchased from OriGene. Full-length OTUD3 WT, full-length C76A mutants and the siRNA-resistant OTUD3 WT were cloned into the pCMV-Myc vectors as indicated. Flag-IRP2 plasmid was gifted by Elizabeth A. Leibold (University of Utah). Antibodies used in immunoblotting are as follows. Anti-IRP2(IRP21-A, 1:1000) and Anti-IRP1(IRP11-A, 1:1000) were purchased from Alpha Diagnostic Intl. Inc. Anti-actin (sc-1616, 1:1000) was from Santa Cruz Biotechnologies. Anti-OTUD3 (HPA028543, 1:100 for immunohistochemistry), anti-Flag (F7425, 1:1000), anti-FPN (SAB1401666, 1:500) and anti-DAT (D6944, 1:1000) were from Sigma. Anti-Myc (M047-3, 1:1000) and anti-HA (M180-3, 1:1000) were from MBL. Anti-ferritin (3998, 1:1000), anti-α-synuclein (2628S, 1:1000), anti-p-α-synuclein (23706, 1:1000) and anti-Iba1(17198, 1:1000) were from Cell Signaling Technology. Anti-TfR1(136800, 1:500) was from Invitrogen Life Technologies. Anti-DMT1 (TA324527, 1:1000) was from origene. Anti-TH (AB152, 1:2000; MAB318, 1:1000) were purchased from Merck Millipore. Anti-p53 (CM5,1:1000) was from Leica. p21 (556430, 1:1000) was from BD Biosciences. Anti-ChAT (A13244, 1:100) was from ABclonal. Anti-GAD67 (20341-1-AP, 1:50) was from Proteintech. To avoid interference of the IgG heavy chain with the Flag-OTUD3 band, Clean-Blot IP Detection Reagents and Kit (Thermo Scientific, 21230) were used.

### Mouse model

The OTUD3 knockout mouse model was generated by Model Animal Research Center of Nanjing University. Strategy of *OTUD3*^−/−^ mouse model was illustrated in our previous study. 24-month-old *OTUD3*^−/−^ mice were applied in the study. A53T transgenic mice (B6; C3-Tg (Prnp-SNCA*A53T)83Vle/J) were originally obtained in breeding pairs from the Jackson Laboratory (004479) to generate a stable breeding colony.

### Subjects and blood sampling

Patients (*n* = 150; 84 men, 66 women; aged 65.93 ± 9.30 years) were recruited from the Department of Neurology, the Affiliated Hospital of Qingdao University. A clinical diagnosis of probable PD was established according to the UK Parkinson’s Disease Society Brain Bank clinical diagnostic criteria. All patients were defined as sporadic, as none of their first-degree relatives had a history of PD. The Hoehn and Yahr staging scale (1–3) was used for the selection of PD cases, all of whom were under therapy at the visit for sampling the blood. Age- and sex-matched healthy controls (*n* = 150; 101 men, 49 women; aged 68.29 ± 8.55 years) were selected from the Physical Examination Center of the Qingdao Municipal Hospital; they were confirmed as healthy and neurologically normal according to their medical history, general examinations, and laboratory testing. The exclusion criteria included prior history of complicating diseases (e.g., pulmonary disease, chronic kidney disease, diabetes mellitus, cancer, or anemia), dementia or other causes of parkinsonian symptoms (e.g., encephalitis, antipsychotic medications, or exposure to toxins), and positive family history for neurodegenerative disorders.

### RNA interference

Smart pool siRNAs against OTUD3 (siRNA 1, 5′-GAAAUCAGGGCUUAAAUGA-3′; siRNA 2, 5′TCGCAAAGGTCACAAACAA-3′) were purchased from Dharmacon. Control siRNAs against OTUD3 were synthesized by the Shanghai Gene Pharm. Transfection was carried out according to the manufacturer’s protocol. After 48 h, cells were washed with phosphate-buffered saline (PBS), lysed directly into TNE lysis buffer (50 mM Tris-HCl pH 7.5, 150 mM NaCl, 1 mM EDTA, and 1% Nonidet P40), and resolved by SDS-polyacrylamide gel electrophoresis (PAGE).

### Cell transfections, immunoprecipitation, and immunoblotting

Cells were purchased from ATCC, authenticated by STR profiling, and tested for mycoplasma contamination by GENEWIZ. Cells were transfected with various plasmids using Lipofectamine 2000 (Invitrogen) reagent according to the manufacturer’s protocol. For immunoprecipitation assays, cells were lysed with HEPES lysis buffer (20 mM HEPES, pH 7.2, 50 mM NaCl, 0.5% Triton X-100, 1 mM NaF, and 1 mM dithiothreitol) supplemented with protease-inhibitor cocktail (Roche). Immunoprecipitations were performed using the indicated primary antibody and protein A/G agarose beads (Santa Cruz) at 4 °C. The immune complexes were then washed with HEPES lysis buffer for four times. Both lysates and immunoprecipitates were examined using the indicated primary antibodies followed by detection with the related secondary antibodies and the Super Signal west pico chemiluminescence substrate (Thermo).

### Protein half-life assay

For the IRP2 half-life assay, Lipofectamine 2000 transfection was performed when HEK293T cells in 2 cm plates reached about 60% confluence. Plasmids encoding OTUD3 or siRNAs were used in transfection as indicated in individual experiments. Twenty-four hours later, the cells were treated with the protein synthesis inhibitor cycloheximide (Sigma, 10 µg/mL) for the indicated durations before collection.

### RT-PCR and quantitative PCR

Total cell RNA was prepared using Trizol reagent (Invitrogen) following the manufacturer’s instructions. Five micrograms of total RNA were subjected to reverse transcription to synthesize cDNA using the First Strand cDNA Synthesis Kit (TOYOBO), and quantitative PCR was performed with the IQ5 system (Bio-Rad). PCRs were performed in 20 μl reaction volumes with SYBR Green PCR master mix (TOYOBO) and 0.2 μM specific primers. The primer sequences used for all qPCRs were described below. IRP2: 5′-CGCCTTTGAGTACCTTATTGAAACA-3′ and 5′-CGTACAGCAGCTTCCAACAAGA-3′; GAPDH: 5′-GCACCGTCAAGGCTGAGAAC-3′ and 5′-TGGTGAAGACGCCAGTGGA-3′.

### Fluorescence microscopy

For the detection of subcellular localization by immunofluorescence, after fixation with 4% paraformaldehyde and permeabilization in 0.2% Triton X-100 (PBS), cells were incubated with the indicated IRP2 and OTUD3 antibodies for 8 h at 4 °C, followed by incubation with TRITC-conjugated or FITC-conjugated secondary antibody (Invitrogen) for 1 h at 25 °C. The nuclei were stained with DAPI (Sigma), and images were visualized with a Zeiss LSM 510 Meta inverted confocal microscope.

### IRP2 ubiquitylation assay

For in vivo IRP2 ubiquitylation assay, Myc-OTUD3, Flag-IRP2 and HA-ubiquitin were transfected into HEK293T cells with Lipofectamine 2000. Twenty-four hours later, the cells were treated with 20 µmol/L of the proteasome inhibitor MG132 (Calbiochem) for 8 h. The cells were washed with PBS, pelleted, and lysed in HEPES buffer plus 0.1% SDS, 20 µmol/L MG132, and protease-inhibitor cocktail. The lysates were centrifuged to obtain cytosolic proteins, incubated with anti-Flag antibody (Abcam) for 3 h, and with protein A/G agarose beads (Santa Cruz) for a further 8 h at 4 °C. The beads were then washed for three times with HEPES buffer. The proteins were released from the beads by boiling in SDS-PAGE sample buffer and analyzed by immunoblotting with anti-HA monoclonal antibody (MBL).

### Rotarod test

Motor coordination and balance were assessed using a rotarod apparatus (Med Associates). The animals were placed on the rolling rods with an initial speed of 4 rpm and an accelerating speed level (4–40 rpm) mode of the apparatus. Then, two trials with an interval trial time of one hour were performed. The mean latency to fall off the rotarod was recorded.

### Pole test

The pole test has been used to detect bradykinesia and motor coordination in PD mice. The mice were placed facing upwards at the top of a wooden pole (50 cm long and 1 cm in diameter) that led into their home cage. The mice were trained to turn to orient downwards and traverse the pole into their home cage. Then, the mice were tested for the time to turn to orient downwards. Five trials were performed with each mouse across the trials. Time was limited to 60 s.

### Catwalk gait analysis

Catwalk XT gait analysis system (Noldus) was used to monitor gait performance. During the test animals were placed in a walkway of 4 cm width and videotaped from below. Footprints were automatically detected by the Catwalk XT 10.0 software. Three compliant runs per animal were recorded and means out of these runs were analyzed over treatment groups with Catwalk XT 10.0 gait analysis software.

### TH immunofluorescence staining

Brains were removed and post-fixed in 4% paraformaldehyde for 6 h, before being transferred to 30% sucrose until sectioning. Sections (20 μm) were cut on a freezing microtome (Leica). Alternate SN sections were stained for TH. After being washed three times in PBS plus 0.3% Triton X-100, sections were incubated overnight with primary antibody of TH at 4 °C. They are then washed three times with PBS and incubated in the second antibody of Alexa Fluor^®^543 donkey anti-mouse IgG at room temperature. Next, sections were rinsed with PBS for three times. Sections were mounted with 70% glycerin. The numbers of TH^+^ in the SN were estimated using MBF Stereo Investigator software (MBF Bioscience). The stereologist was blind to the treatment received. One of every six serial sections of the mid-brain was counted to cover the entire mouse. Stereological details were as follows: counting grid, 240 × 180 μm; counting frames, 80 × 60 μm.

### High-performance liquid chromatography with electrochemical detection (HPLC-ECD)

One side of the striatum was isolated carefully and transferred to liquid nitrogen. Samples were weighed and then homogenized in 0.3 ml liquid A (0.4 M perchloric acid). After initial centrifugation (12,000 rpm for 20 min at 4 °C), 80 μl of the supernatant was transferred into Eppendorf tubes, and 40 μl liquid B (20 mM citromalic acid potassium, 300 mM dipotassium phosphate, 2 mM ethylene diaminetetraacetic acid (EDTA)·2Na) were added. After additional centrifugation (12,000 rpm for 20 min at 4 °C), 100 μl of the supernatant was assayed for DA by HPLC. Separation was achieved on a PEC18 reversed-phase column. The mobile phase (20 mM citromalic acid, 50 mM sodium caproate, 0.134 mM EDTA·2Na, 3.75 mM sodium octane sulfonic acid, 1 mM di-sec-butylamine and containing 5% (v/v) methanol) was used at a flow-rate of 1 mL/min. A 2465 electrochemical detector (Waters) was employed and operated in screen mode.

### Iron content measurements

Inductively coupled plasma mass spectrometer (ICP-MS) 7500CE (Agilent) was used for the determination of the iron content of different tissues. Before the measurement, samples were digested in order to destruct tissues and proteins, and obtain a limpid solution. Two milliliter aliquot of nitric acid (65%) and 1.5 ml of hydrogen peroxide (35%) were added. The mixture was placed in a Milestone MS-200 microwave oven and exposed at 250 W for 2 min, 0 W for 2 min, then 250 W for 6 min and 650 W for 5 min and 5 min of ventilation. After 20 min of cooling at room temperature, samples were ready for iron content determinations.

### Assays with MEFs

MEFs were isolated from embryonic day (E) 12.5 embryos and cultured in alpha-MEM medium supplemented with 10% FBS, penicillin/streptomycin using standard techniques.

### Flow cytometric measurement of ROS and Δψm

Flow cytometry (Becton Dickinson) was used to measure the changes of ROS and Δψm. Cells were washed with HBS for three times followed by incubation with 5 μM 2′,7′-dichlorofluorescein diacetate (H_2_DCFDA) (Molecular Probes) or 5 μM Rhodamine123 (Sigma) for 30 min at 37 °C in dark. After being washed three times with HBS, labeled cells were resuspended in 1 ml HBS. For analysis, 488 nm excitation and 525 nm emission wavelengths were used to assess 10,000 cells for each group. Fluorescence values of the control were normalized to 100%. The results were expressed as the percentage of fluorescence intensity for each experimental group relative to the control.

### Apoptosis assays

The cells were washed with PBS and stained with fluorescein isothiocyanate Annexin V and propidium iodide according to the manufacturer’s protocol (BD Bioscience). Apoptotic cells (Annexin V positive, propidium iodide negative) were then determined by flow cytometry.

### Adenosine triphosphate (ATP) levels and pyruvate contents analyses

The intracellular ATP and pyruvate contents were measured using ATP assay kit (BC0305, Solarbio) and pyruvate assay kit (BC2205, Solarbio), respectively. For measurement, 20 mg SN tissues from 2 mice were mixed as a sample, lysed using tissue extract in pyruvate assay kit, and measured according to the manufacturer’s protocol.

### Sanger sequencing

Genomic DNA was extracted from blood cells of each case of PD and control group. DNA quality and concentration were measured by 1.0% agarose gel electrophoresis and UV spectrometry on a NanoDrop 2000 device, respectively. The fragments were selected by agarosegel electrophoresis, and PCR was performed for enrichment. The produced library was sequenced with the ABI3730xl DNA Analyzer (the Beijing Genomics Institute, BGI). The phred\phrap was used to analysis data.

### Statistical analysis

All statistical analyses were performed in SPSS 19.0 (IBM) and GraphPad Prism 7. For quantification of IHC on IRP2, TfR1 and Ferritin, the tissues were analyzed by quantifying at least 5 images at least 100 × 100 μm area using ImageJ software. Quantification analysis of western blots was performed in ImageJ. Data were expressed as mean values ± S.E.M. Detailed *n* values for each panel in the figures were stated in the corresponding legends. Independent sample t-test (for two group comparisons) or One-way analysis of variance (ANOVA) followed by Dunn’s multiple comparison tests (for more than two group comparisons) were used for statistical analyses. All statistical tests were two-tailed, and *p* < 0.05 was considered to be statistically significant. In the mouse experiments, the mice were randomly assigned and the investigators were blinded to experiments and outcome assessment.

## Supplementary information

Checklist

Author Contribution form

Supplementary Information

Supplementary Table 1

Supplementary Table 2

Uncropped Western Blots

Source Statistics Data

## Data Availability

All data are included within the article, supplementary information, or available from the authors upon reasonable request.
