# Supplementary material for: Deubiquitylase OTUD3 prevents Parkinson’s disease through stabilizing iron regulatory protein 2
Source: Cell Death Dis. 2022 Apr 30;13(4):418. doi: 10.1038/s41419-022-04704-0 (PMC9056525; doi:10.1038/s41419-022-04704-0)
Supplement: Supplementary file 2 — Author Contribution form [file 41419_2022_4704_MOESM2_ESM.pdf]

DECLARATION OF CONTRIBUTIONS TO ARTICLE

ADMC

Manuscript Number: Manuscript CDDIS-21-3623

Journal Name: *Cell Death & Disease* (the 'Journal')

Proposed Title of the Contribution:

Deubiquitylase OTUD3 prevents Parkinson's disease through stabilizing iron regulatory protein 2 (the 'Contribution)

Author(s):

Fengju Jia, Hongchang Li, Qian Jiao, Chaonan Li, Lin Fu, Chunping Cui, Hong Jiang, Lingqiang Zhang (the 'Authors')

For all *CDDis* articles, each person named as an author in the published version must be able to show he or she has contributed substantially to the article.

Authorship credit should be based on 1) substantial contributions to conception and design, acquisition of data, or analysis and interpretation of data; 2) drafting the article or revising it critically for important intellectual content; and 3) final approval of the version to be published. Authors should meet conditions 1, 2 and 3.

Any person who cannot be shown to have made a substantial contribution to the article cannot be listed as an author in the final version. The name of any person who is deemed to have made a minor contribution can, however, appear in the Acknowledgments section of the article.

Please complete the table below to indicate the contributions of all named authors to the manuscript.

Author Full Name:	Specification of Contribution to the Manuscript:
Fengju Jia	performed the majority of experiments and write the manuscript.
Hongchang Li	generated OTUD3-/- mice and MEFs, contributed to phenotype analysis.
Qian Jiao	analyzed the data.
Chaonan Li	analyzed the data.
Lin Fu	analyzed the data.
Chunping Cui	analyzed the data.
Hong Jiang	conceived and designed research, and helped write the manuscript.
Lingqiang Zhang	conceived and designed research, and helped write the manuscript.

Please complete the table below to indicate the contributions of all named authors to the figures.

Figure 1:

Fengju Jia: generated the data and prepared panel a, b, c, d, e, f, g, j, k, n.
Qian Jiao: generated the data and prepared panel h, i.
Hongchang Li: generated the data and prepared panel l.
Chunping Cui: generated the data and prepared panel m and assembled the figure.

Figure 2:

Fengju Jia: generated the data and prepared panel a, b, c, d, e, f.
Hongchang Li: generated the data and prepared panel g, h, k and assembled the figure.
Chaonan Li: generated the data and prepared panel i, j, k.

Figure 3:

Fengju Jia: generated the data and prepared panel a, b, c, d, e, f.
Hongchang Li: generated the data and prepared panel g, h, i, j, k and assembled the figure.

Figure 4:

Fengju Jia: generated the data and prepared panel a, b, d.
Hongchang Li: generated the data and prepared panel c and assembled the figure.

Figure 5:

Hongchang Li: generated the data a, b, c, d, e and assembled the figure.

Figure 6:

Fengju Jia: generated the data a, b, c, d.
Lin Fu: analyzed the data a, b, c, d.

Signed for and on behalf of the Author(s):	Print Name:	Date:
	Lingqiang Zhang	2022/2/13
