# Supplementary material for: Deubiquitylase OTUD3 prevents Parkinson’s disease through stabilizing iron regulatory protein 2
Source: Cell Death Dis. 2022 Apr 30;13(4):418. doi: 10.1038/s41419-022-04704-0 (PMC9056525; doi:10.1038/s41419-022-04704-0)
Supplement: Supplementary file 3 — Supplementary Information [file 41419_2022_4704_MOESM3_ESM.doc]

Supplementary Materials for

**Deubiquitylase OTUD3 prevents Parkinson’s disease through stabilizing iron regulatory protein 2**

Fengju Jia, Hongchang Li, Qian Jiao, Chaonan Li, Lin Fu, Chunping Cui, Hong Jiang and Lingqiang Zhang

Correspondence to: hongjiang@qdu.edu.cn/zhanglq@nic.bmi.ac.cn

**This PDF file includes: Supplementary Fig. 1 to Fig. 8**


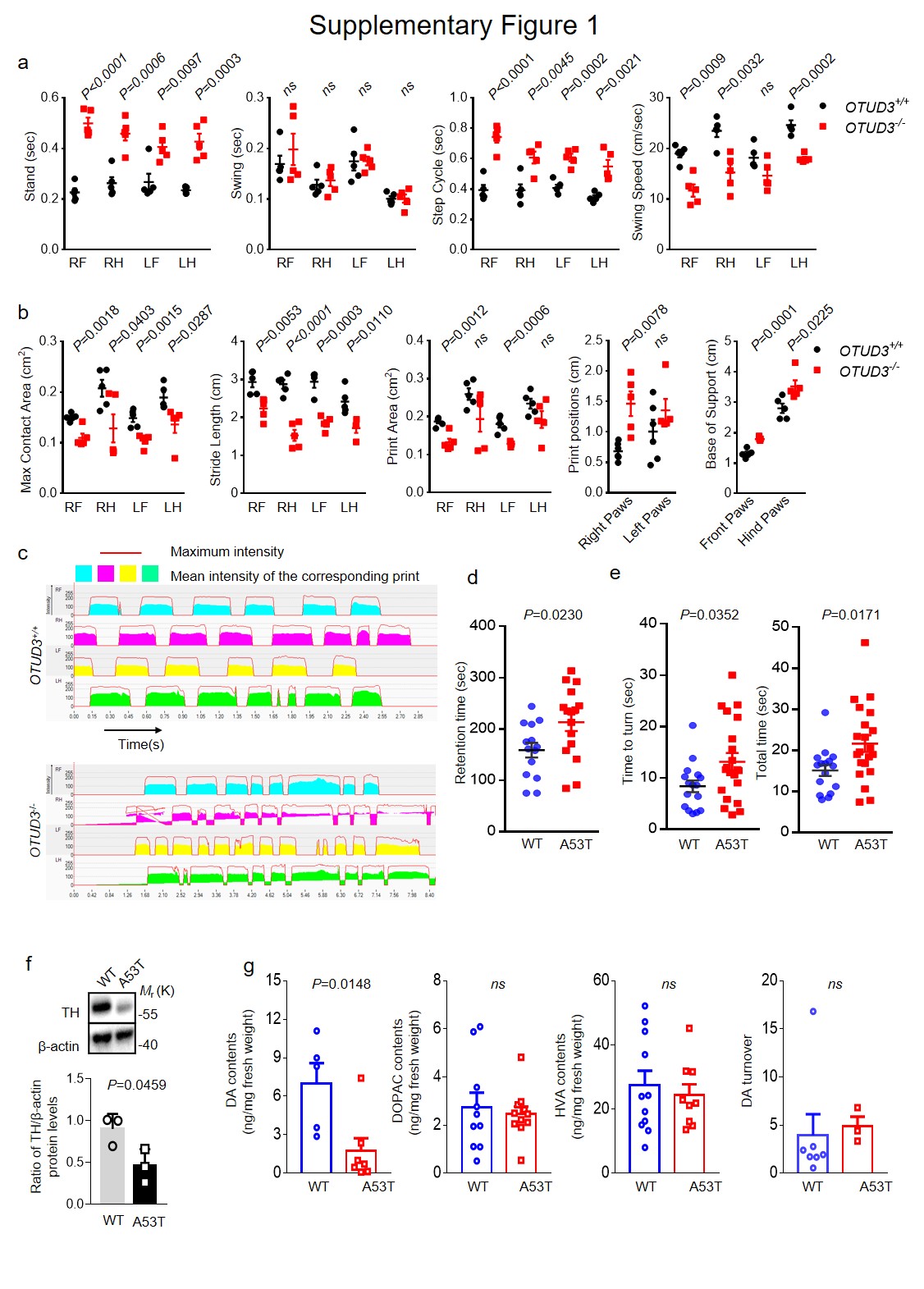


**Supplementary figure 1 *OTUD3*^-/-^ mice resembled the symptoms of PD. a** Dynamic paw parameters were affected in *OTUD3*^-/-^ mice. Stand was the duration in seconds of contact while swing was the duration in seconds of no contact of a paw with the glass plate. Therefore, step cycle represented the sum of swing and stand duration. *OTUD3*^+/+^: *n*=5; *OTUD3*^-/-^: *n*=5. **b-c** Static paw parameters of *OTUD3*^+/+^ (*n*=5) and *OTUD3*^-/-^ (*n*=5) mice were analyzed. Print area: the area of contact of a paw with the glass plate. Stride length: the distance between initial contacts of the same paw in one complete stride. **d** Rotarod test was used to measure the residence time of mice on rotarod treadmills. WT: *n*=14; A53T: *n*=16. **e** The time to turn to orient downwards and total time were assessed in the pole test. WT: *n*=16; A53T: *n*=21. **f** TH protein levels were evaluated in SN of A53T transgenic mice. WT: *n*=3; A53T: *n*=3. **g** DA contents and its metabolites in the striatum were determined by HPLC-ECD (for DA measured: WT: *n*=5, A53T: *n*=7; for DOPAC measured: WT: *n*=10, A53T: *n*=11; for HVA measured: WT: *n*=11, A53T: *n*=9; for HVA tested: WT: *n*=7, A53T: *n*=3). All panels are representative results of three or more independent experiments. Data are depicted as bar graphs with mean ± s.e.m. The statistics data and uncropped western blots can be found in Supplemental Material.


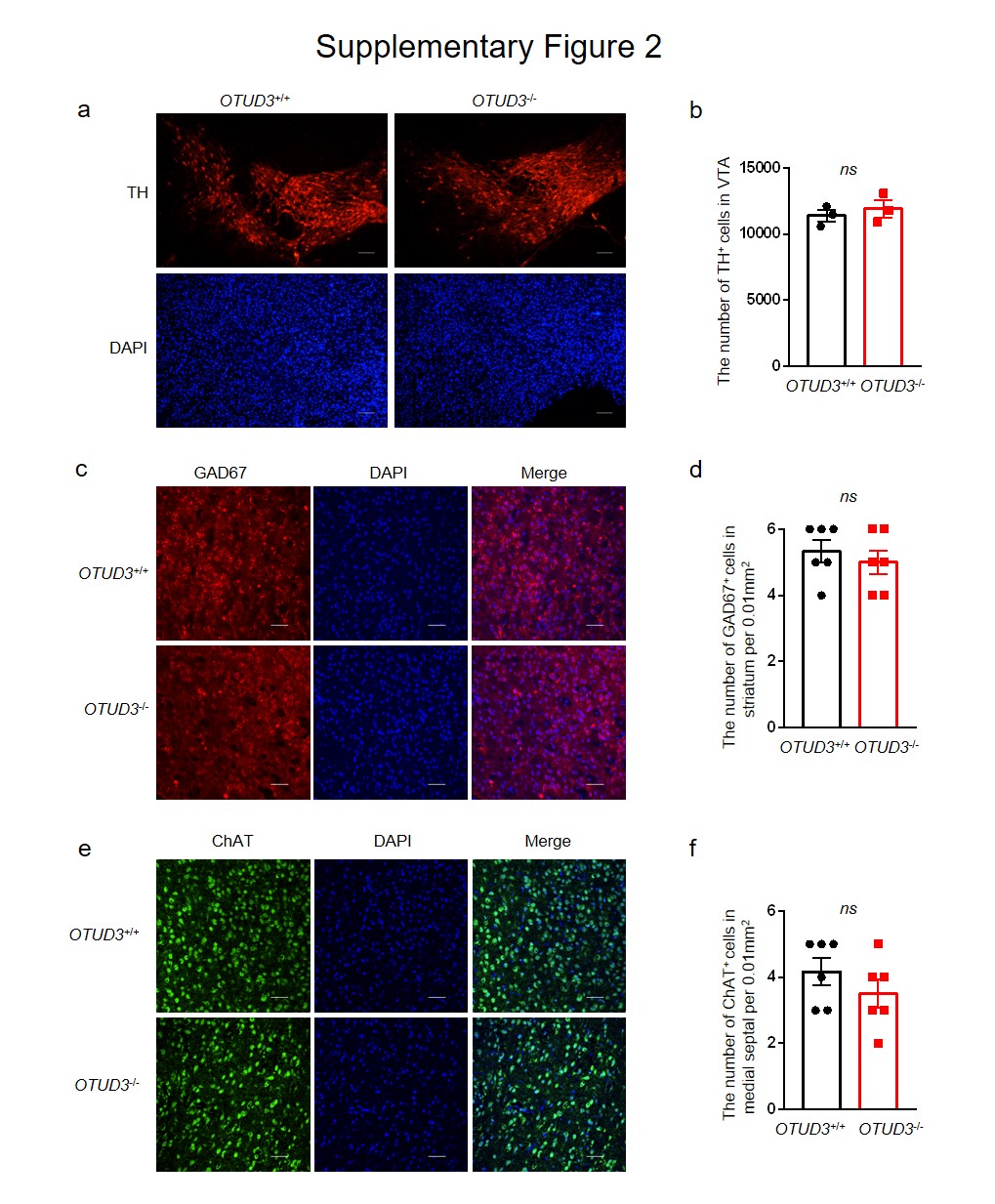


**Supplementary figure 2 OTUD3 deletion did** **not affect DA neurons in VTA, GABAergic neurons in striatum and cholinergic neurons in MS. a** Representative imaging of TH-positive neurons in VTA. Scale bars, 100 µm. **b** The quantification of TH-positive neurons in VTA of indicated mice. *OTUD3*^+/+^: n=3; *OTUD3*^-/-^: n=3. **c** Representative imaging of GAD67-positive neurons in striatum. Scale bars, 100 µm. **d** The quantification of GAD67-positive neurons in striatum of indicated mice. *OTUD3*^+/+^: n=3; *OTUD3*^-/-^: n=3. **e** Representative imaging of GAD67-positive neurons in striatum. Scale bars, 100 µm. **f** The quantification of ChAT-positive neurons in MS of indicated mice. *OTUD3*^+/+^: n=3; *OTUD3*^-/-^: n=3. Data are depicted as bar graphs with mean ± s.e.m. Student’s *t*-test. The statistics data can be found in Supplemental Material.


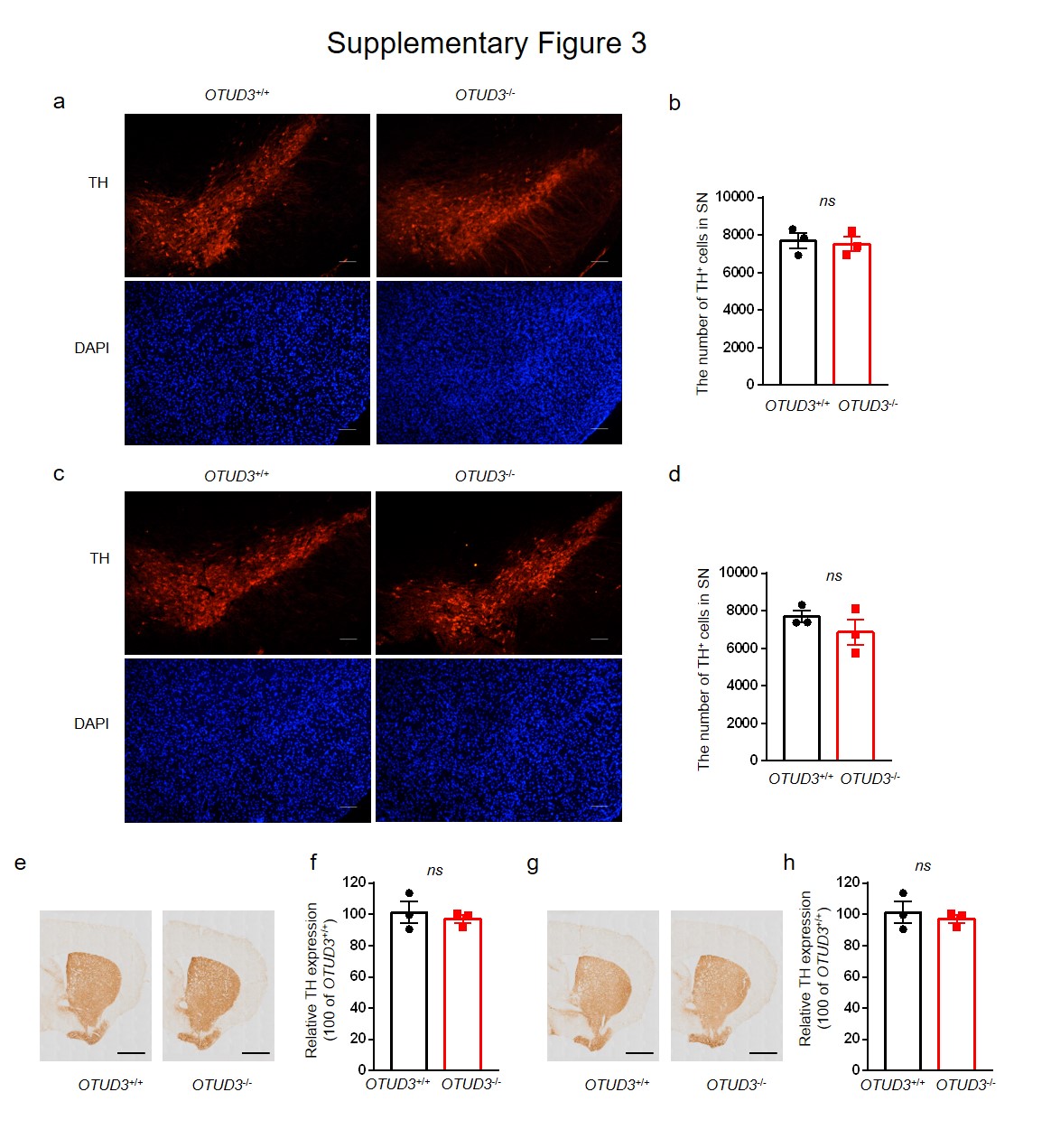


**Supplementary figure 3 OTUD3 deletion caused age-dependent dopaminergic neurons lesions. a** Representative imaging of TH-positive neurons in SN of young mice (1.5 months old). Scale bars, 100 µm. **b** The quantification of TH-positive neurons in SN of young mice. *OTUD3*^+/+^: n=3; *OTUD3*^-/-^: n=3. **c** Representative imaging of TH-positive neurons in SN of adult mice (3 months old). Scale bars, 100 µm. **d** The quantification of TH-positive neurons in SN of adult mice. *OTUD3*^+/+^: n=3; *OTUD3*^-/-^: n=3. **e** Representative imaging of TH intensity in the striatum of young mice. Scale bars, 1 mm. **f** The quantification of TH intensity the striatum of young mice. *OTUD3*^+/+^: n=3; *OTUD3*^-/-^: n=3. **g** Representative imaging of TH intensity in the striatum of adult mice. Scale bars, 1 mm. **h** The quantification of TH intensity in the striatum of adult mice. *OTUD3*^+/+^: n=3; *OTUD3*^-/-^: n=3. Data are depicted as bar graphs with mean ± s.e.m. Student’s *t*-test. The statistics data can be found in Supplemental Material.


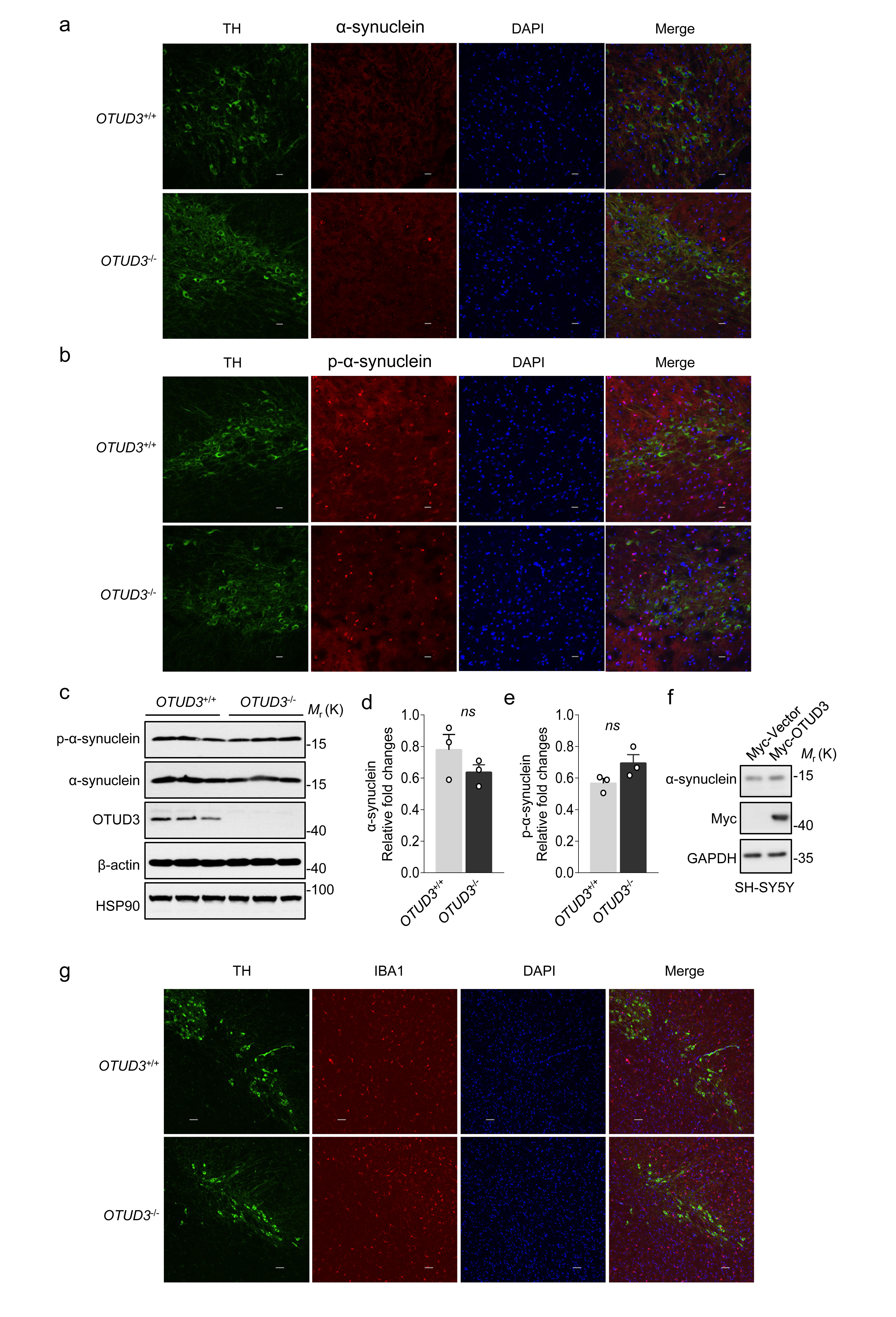


**Supplementary figure 4 OTUD3 deletion did not exhibit pathologically fibrillary form of α-synuclein and activation of microglial cells in SN of 24 months old mice. a** Representative imaging of α-synuclein in SN. Scale bars, 100 µm. **b** Representative imaging of phosphorylated α-synuclein in SN. Scale bars, 100 µm. **c** The levels of α-synuclein and phosphorylated α-synuclein in SN of young mice. *OTUD3*^+/+^: n=3; *OTUD3*^-/-^: n=3. **d** Quantification of α-synuclein relative to β-actin protein levels for Sup Fig. 2c. *OTUD3*^+/+^: n=3; *OTUD3*^-/-^: n=3. **e** Quantification of phosphorylated α-synuclein relative to α-synuclein protein levels for Sup Fig. 2c. *OTUD3*^+/+^: n=3; *OTUD3*^-/-^: n=3. **f** α-synuclein protein levels were measured in SH-SY-5Y cells with overexpression of OTUD3. **g** Representative imaging of Iba1-positive neurons in SN. Scale bars, 100 µm. Data are depicted as bar graphs with mean ± s.e.m. Student’s *t*-test. The statistics data and uncropped western blots can be found in Supplemental Material.


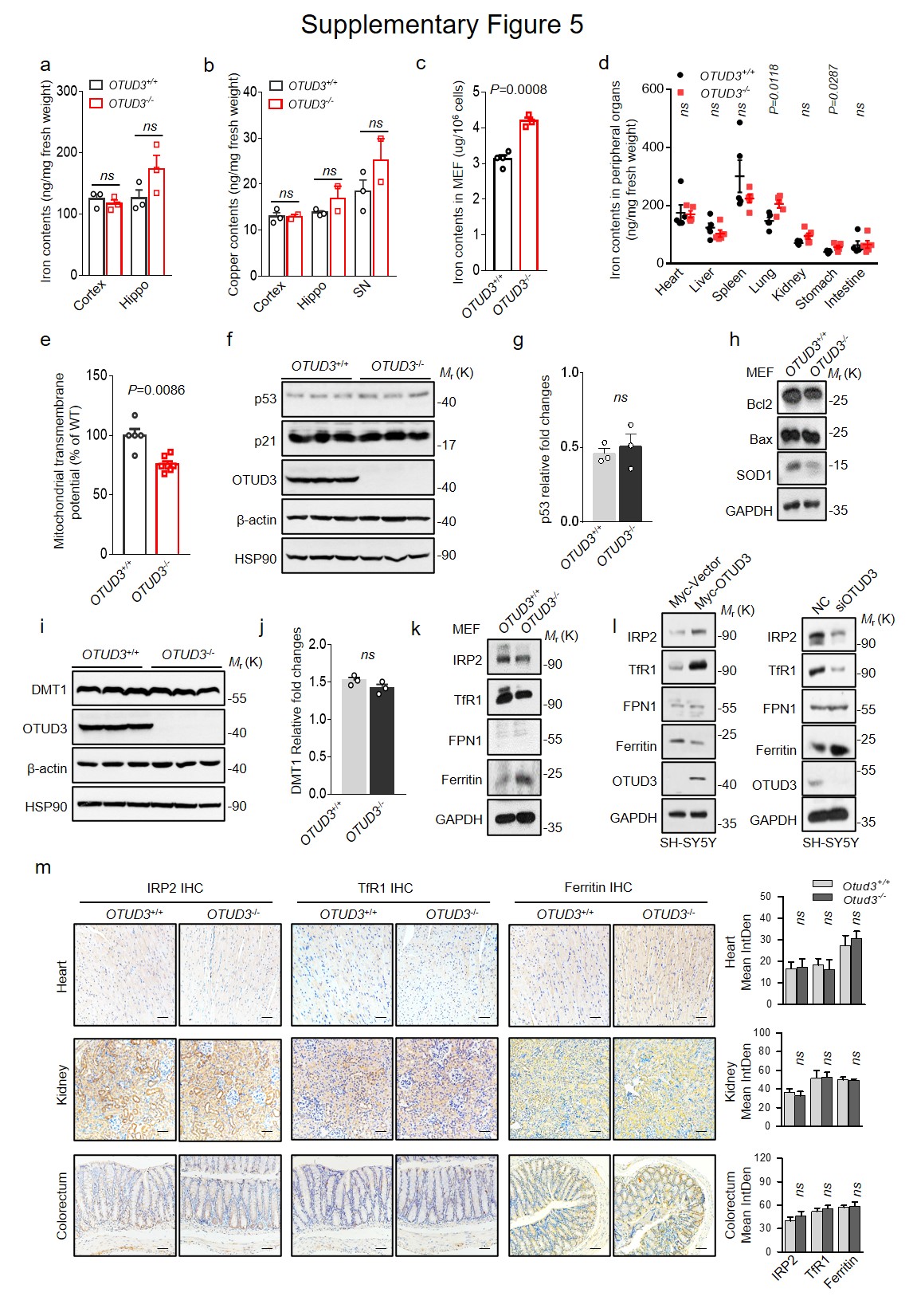


**Supplementary figure 5 OTUD3 deletion induced disorder of iron metabolism. a** Iron contents were detected in the cortex and hippocampus of indicated mice. *OTUD3*^+/+^: *n*=3; *OTUD3*^-/-^: *n*=3. **b** Copper contents were evaluated in the SN, cortex and hippocampus. *OTUD3*^+/+^: *n*=3; *OTUD3*^-/-^: *n*=2. **c, d** Iron contents were detected in MEFs and peripheral tissues of *OTUD3*^+/+^ and *OTUD3*^-/-^ mice (for MEF: *OTUD3*^+/+^: *n*=4, *OTUD3*^-/-^: *n*=3; for peripheral tissues: *OTUD3*^+/+^: *n*=5, *OTUD3*^-/-^: *n*=5). **e** Mitochondrial transmembrane potential (ΔΨm) was measured in MEFs. *OTUD3*^+/+^: *n*=5; *OTUD3*^-/-^: *n*=7. **f** p53 protein levels were evaluated in SN. **g** Quantification of the p53 protein relative to β-actin protein levels. **h** The levels of superoxide dismutase 1 (SOD1) and Bcl-2 were also observed. **i** DMT1 protein levels were evaluated in SN. **j** Quantification of the DMT1 protein relative to β-actin protein levels. **k** Changes of iron transport-related proteins in MEFs. **l** The effects of overexpression and knock-down of OTUD3 on iron transport-related proteins in SH-SY-5Y cells. **m** The expression of iron transport-related proteins in various organs of *OTUD3*^-/-^ mice by immunohistochemistry assay. Scale bars, 50 μm. All panels are representative results of three or more independent experiments. Data are depicted as bar graphs with mean ± s.e.m. Student’s *t*-test. The statistics data and uncropped western blots can be found in Supplemental Material.


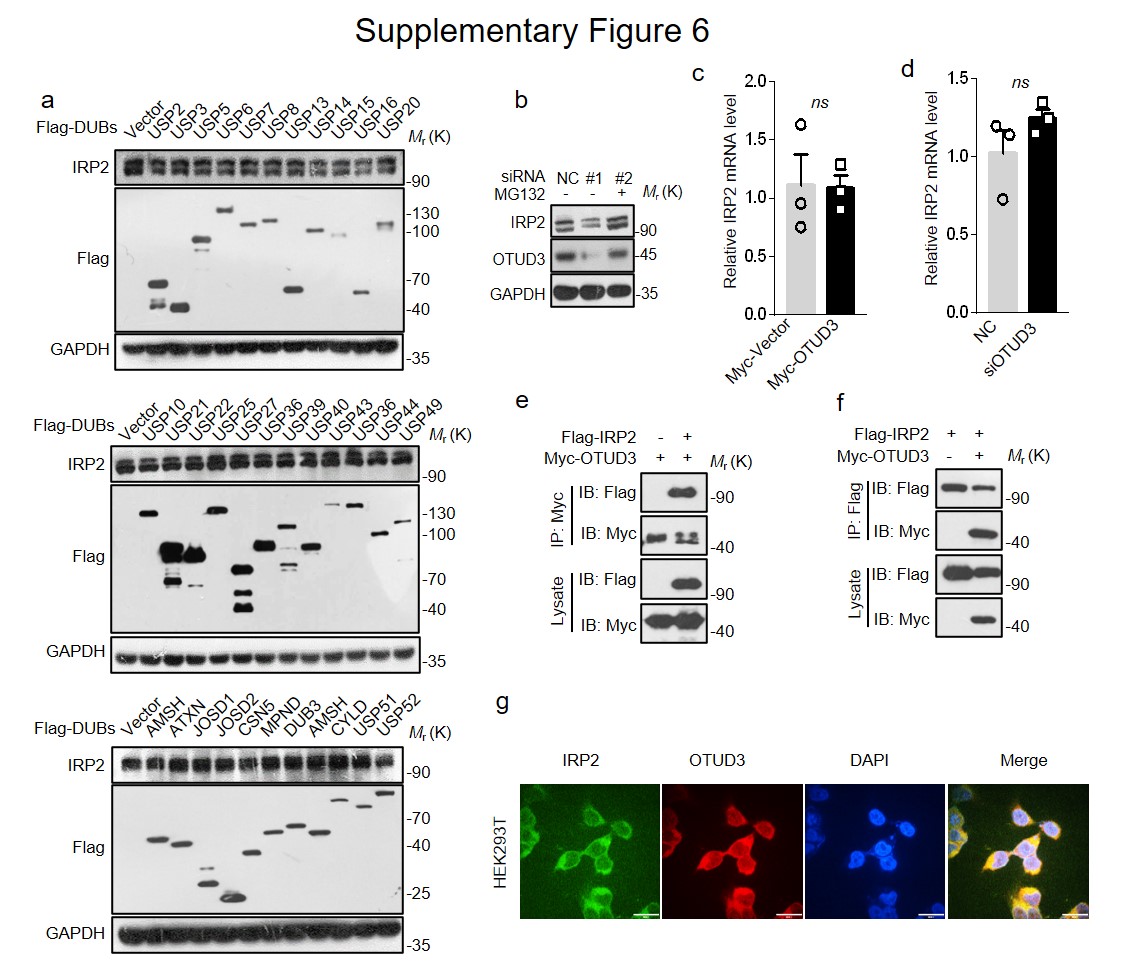


**Supplementary figure 6 OTUD3 maintained IRP2 stability. a** Protein level analysis of IRP2 in the presence or absence of overexpressed the indicated DUBs. **b** HEK293T cells transfected with the indicated siRNA were treated with or without the proteasome inhibitor MG132 (20 µM, 8h). **c, d** Overexpression of OTUD3 or siRNAs had no significant effects on IRP2 mRNA levels in HEK293T cells. Data shown are mean ± s.e.m. *n*=3 independent experiments. Data were analysed using Student’s *t*-test. **e, f** HEK293T cells transfected with the indicated constructs were subject to immunoprecipitation with anti-Myc or anti-Flag antibodies. **g** HEK293T cells were fixed and stained with anti-IRP2 (green) and anti-OTUD3 (red). Nuclei were counter stained with DAPI (blue). Scale bars, 20 μm. The statistics data and uncropped western blots can be found in Supplemental Material.

**
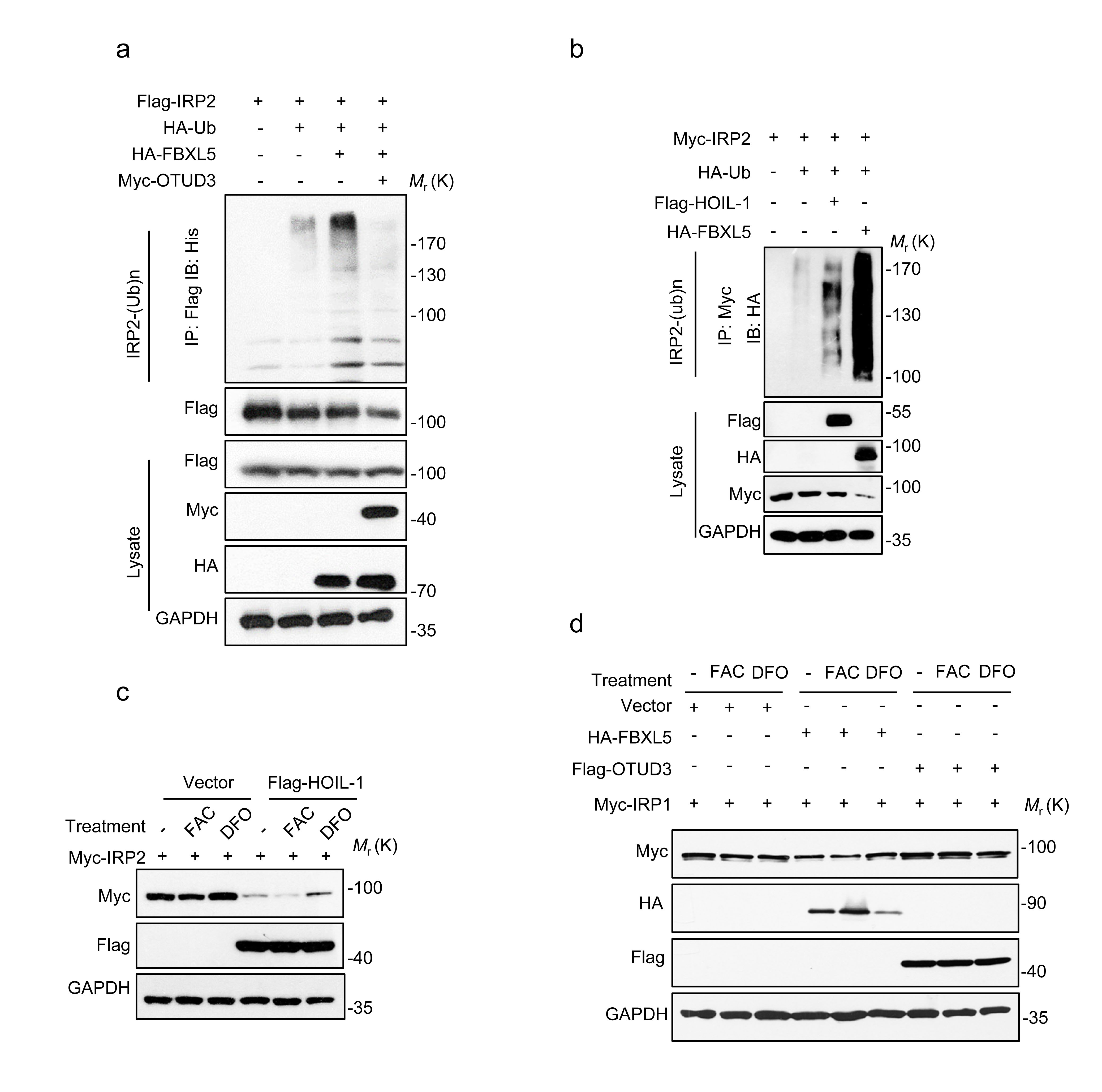
**

**Supplementary figure 7 OTUD3 de-polyubiquitylated IRP2**. **a, b** IRP2 ubiquitylation was analysed in cells transfected with FBXL5 or HOIL-1 together with OTUD3 or not. **c, d** HEK293T cells transfected with the indicated plasmids were treated by 100 µg/ml FAC or 100 µg/ml DFO, and then the expression levels of related proteins were analyzed. All panels are representative results of three or more independent experiments. The uncropped western blots can be found in Supplemental Material.


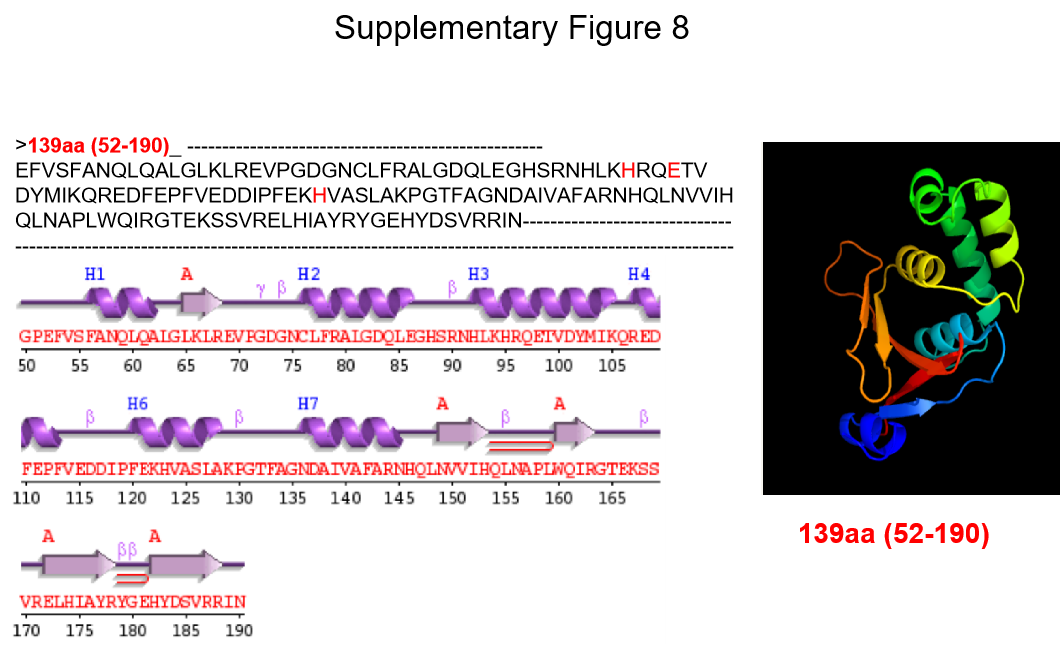


**Supplementary figure 8** The PDB formatted model of OTU domain of OTUD3.
