# Supplementary material for: Deubiquitylase OTUD3 prevents Parkinson’s disease through stabilizing iron regulatory protein 2
Source: Cell Death Dis. 2022 Apr 30;13(4):418. doi: 10.1038/s41419-022-04704-0 (PMC9056525; doi:10.1038/s41419-022-04704-0)
Supplement: Supplementary file 4 — Supplementary Table 1 [file 41419_2022_4704_MOESM4_ESM.docx]

**Table 1. Confirmation of inconsistent genotypes detected using Sangersequencing.**

| **SNP ID** | **Exon location** | **Sample name** | **SNP site base** | **Nucleic acid changes** | **Protein changes** | **Reference sequence base** | **Mutation site region sequence** |
| --- | --- | --- | --- | --- | --- | --- | --- |
| rs75742716 | OTUD3-Exon6 | PD62 | TG | c.810T>G | p.Leu270= | T | ATTGCCGTGCT[T/G]CGGATGAACC |
| / | OTUD3-Exon7 | PD8 | CA | c.1008C>A | p.N336K | C | CAAATAAAAA[A/C]CAGCTCGCAA |
| rs2298110 | OTUD3-Exon7 | Control41 | AG | c.962A>G | p.N321S | A | ACCGAAAACA[A/G]TAAGGCACAG |
|  |  | Control45 | AG | c.962A>G | p.N321S | A | ACCGAAAACA[A/G]TAAGGCACAG |
|  |  | Control79 | AG | c.962A>G | p.N321S | A | ACCGAAAACA[A/G]TAAGGCACAG |
|  |  | Control81 | AG | c.962A>G | p.N321S | A | ACCGAAAACA[A/G]TAAGGCACAG |
|  |  | Control98 | AG | c.962A>G | p.N321S | A | ACCGAAAACA[A/G]TAAGGCACAG |
|  |  | PD21 | AG | c.962A>G | p.N321S | A | ACCGAAAACA[A/G]TAAGGCACAG |
|  |  | PD73 | AG | c.962A>G | p.N321S | A | ACCGAAAACA[A/G]TAAGGCACAG |
|  |  | PD125 | AG | c.962A>G | p.N321S | A | ACCGAAAACA[A/G]TAAGGCACAG |
| rs10916668 | OTUD3-Exon7 | Control41 | AG | c.997G>A | p.A333T | G | AGAAAACAAA[A/G]CAAATAAAAA |
|  |  | Control45 | AG | c.997G>A | p.A333T | G | AGAAAACAAA[A/G]CAAATAAAAA |
|  |  | Control79 | AG | c.997G>A | p.A333T | G | AGAAAACAAA[A/G]CAAATAAAAA |
|  |  | Control81 | AG | c.997G>A | p.A333T | G | AGAAAACAAA[A/G]CAAATAAAAA |
|  |  | Control98 | AG | c.997G>A | p.A333T | G | AGAAAACAAA[A/G]CAAATAAAAA |
|  |  | PD21 | AG | c.997G>A | p.A333T | G | AGAAAACAAA[A/G]CAAATAAAAA |
|  |  | PD73 | AG | c.997G>A | p.A333T | G | AGAAAACAAA[A/G]CAAATAAAAA |
|  |  | PD125 | AG | c.997G>A | p.A333T | G | AGAAAACAAA[A/G]CAAATAAAAA |
| rs78466831 | OTUD3-Exon7 | Control65 | AG | c.863G>A | p.G288D | G | GAGCCCAGTG[A/G]TCGAGTGCTG |
|  |  | PD65 | AG | c.863G>A | p.G288D | G | GAGCCCAGTG[A/G]TCGAGTGCTG |
|  |  | PD78 | AG | c.863G>A | p.G288D | G | GAGCCCAGTG[A/G]TCGAGTGCTG |
|  |  | PD79 | AG | c.863G>A | p.G288D | G | GAGCCCAGTG[A/G]TCGAGTGCTG |
