# Supplementary material for: Deubiquitylase OTUD3 prevents Parkinson’s disease through stabilizing iron regulatory protein 2
Source: Cell Death Dis. 2022 Apr 30;13(4):418. doi: 10.1038/s41419-022-04704-0 (PMC9056525; doi:10.1038/s41419-022-04704-0)
Supplement: Supplementary file 5 — Supplementary Table 2 [file 41419_2022_4704_MOESM5_ESM.docx]

**Table 2. Summary statistics for the association of single nucleotide polymorphisms of OTUD3 with PD.**

| **Genetic models** | **Controls(*n*=150)** | **PD(*n*=150)** | ***P* value** |
| --- | --- | --- | --- |
| rs75742716 Codominant model | | | |
| TT | 150 | 149 |  |
| TG | 0 | 1 | 1.000 |
| c.1008C>A Codominant model | |  |  |
| CC | 150 | 149 |  |
| CA | 0 | 1 | 1.000 |
| rs2298110 Codominant model | |  |  |
| AA | 145 | 147 |  |
| AG | 5 | 3 | 1.000 |
| rs10916668 Codominant model | |  |  |
| AA | 145 | 147 |  |
| AG | 5 | 3 | 1.000 |
| rs78466831 Codominant model | |  |  |
| AA | 149 | 147 |  |
| AG | 1 | 3 | 1.000 |
