# Supplementary material for: Deubiquitylase OTUD3 prevents Parkinson’s disease through stabilizing iron regulatory protein 2
Source: Cell Death Dis. 2022 Apr 30;13(4):418. doi: 10.1038/s41419-022-04704-0 (PMC9056525; doi:10.1038/s41419-022-04704-0)

Fig 1l.

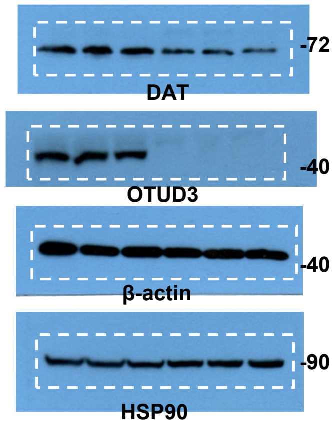

Fig 1n.

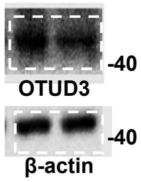

Fig 2f.

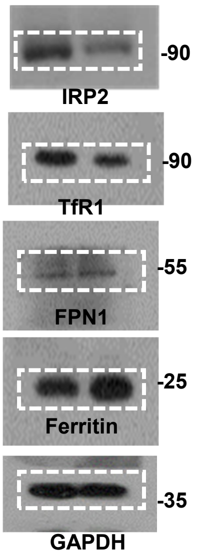

Fig 2g.

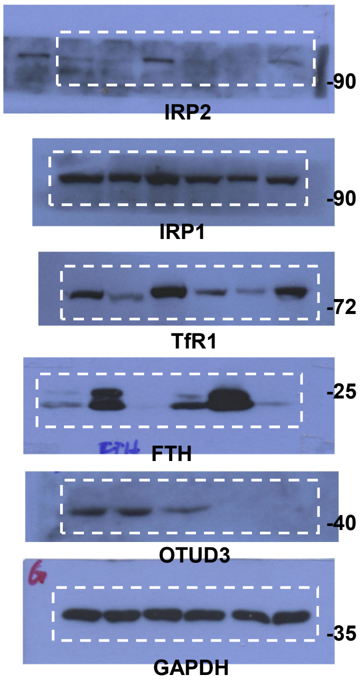

Fig 2h.

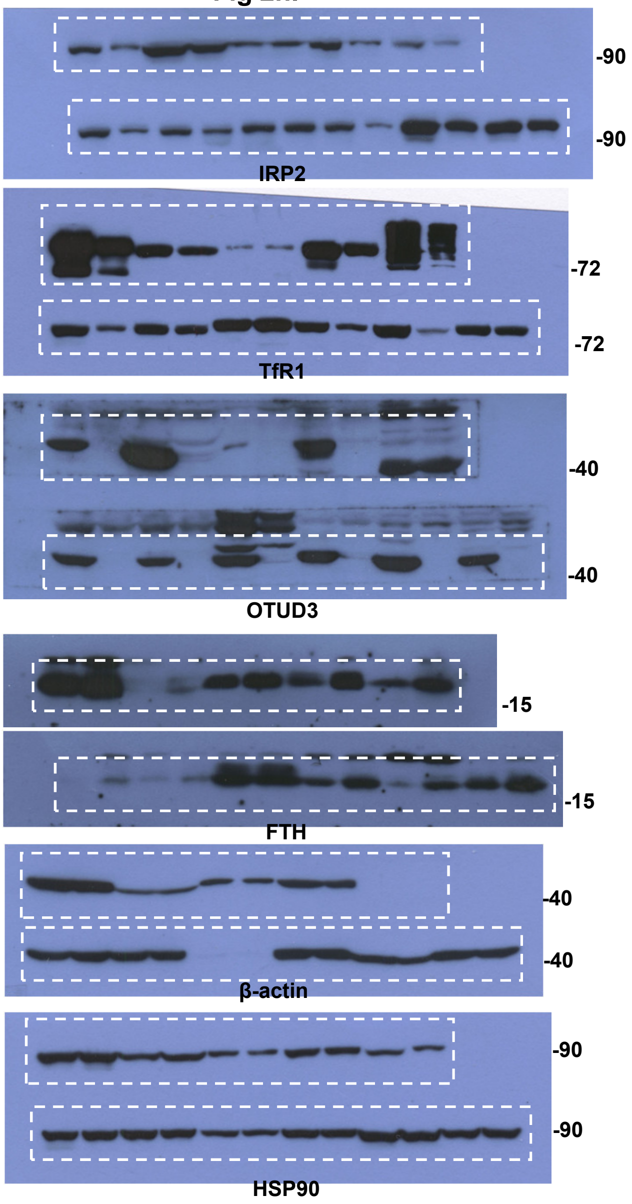

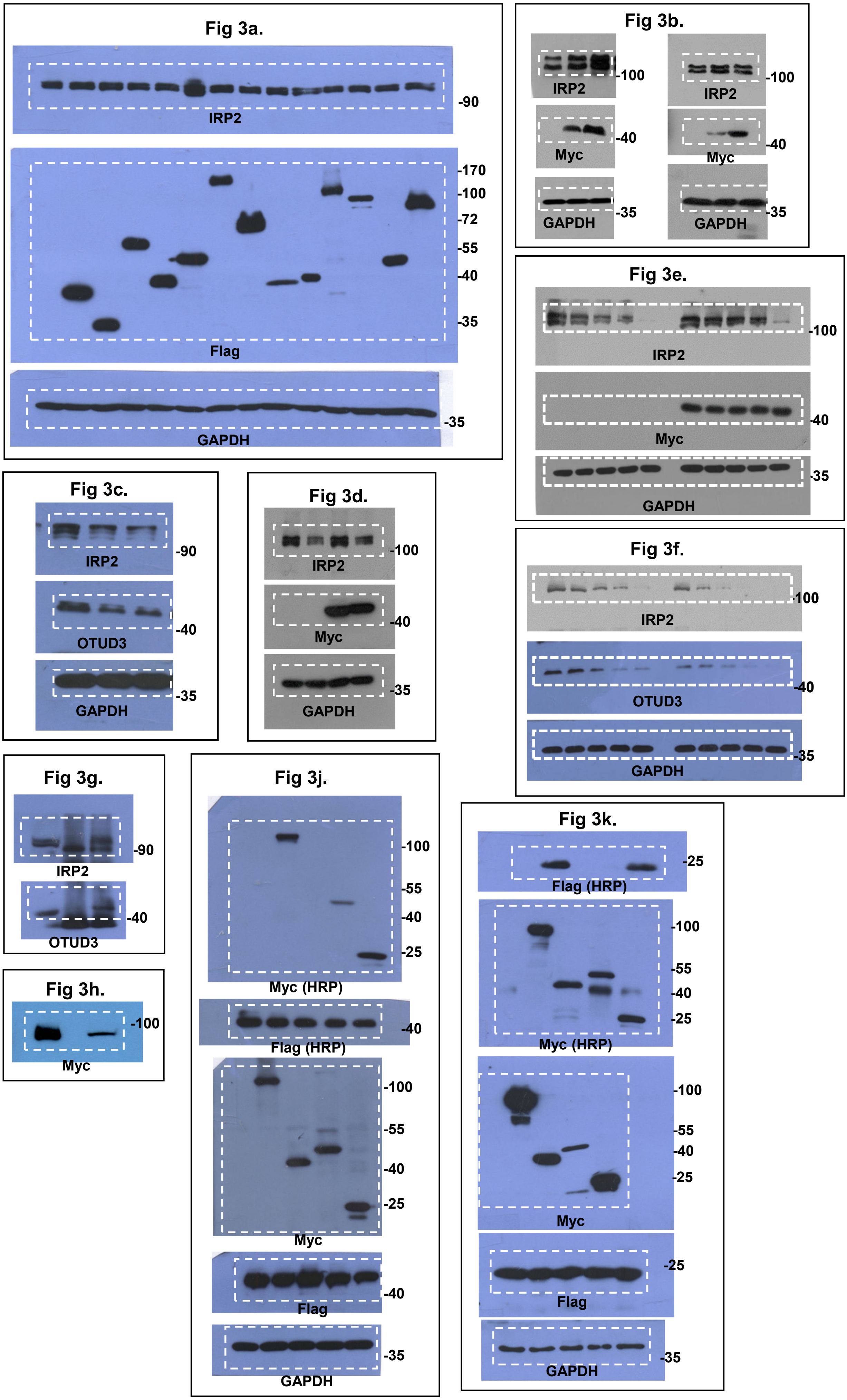
Fig 3a.
IRP2
-90
Flag
-170
-100
-72
-55
-40
-35
GAPDH
-35
Fig 3b.
IRP2
-100
Myc
-40
GAPDH
-35
IRP2
-100
Myc
-40
GAPDH
-35
Fig 3e.
IRP2
-100
Myc
-40
GAPDH
-35
Fig 3c.
IRP2
-90
OTUD3
-40
GAPDH
-35
Fig 3d.
IRP2
-100
Myc
-40
GAPDH
-35
Fig 3f.
IRP2
-100
OTUD3
-40
GAPDH
-35
Fig 3g.
IRP2
-90
OTUD3
-40
Fig 3h.
Myc
-100
Fig 3j.
Myc (HRP)
-100
-55
-40
-25
Flag (HRP)
-40
Myc
-100
-55
-40
-25
Flag
-40
GAPDH
-35
Fig 3k.
Flag (HRP)
-25
Myc (HRP)
-100
-55
-40
-25
Myc
-100
-55
-40
-25
Flag
-25
GAPDH
-35

Fig 4a.

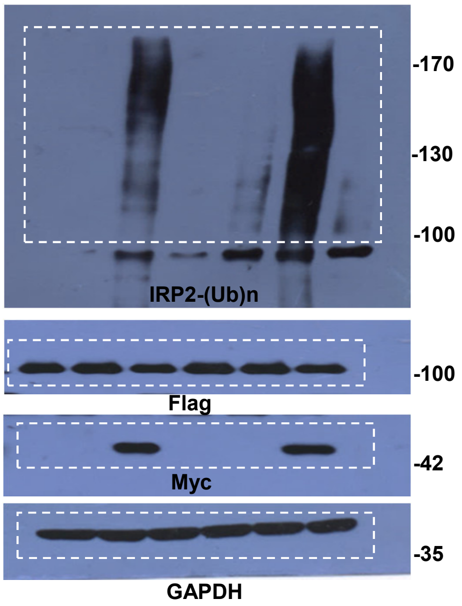

Fig 4b.

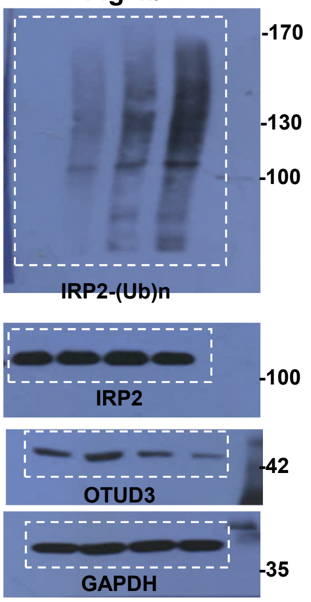

Fig 4c.

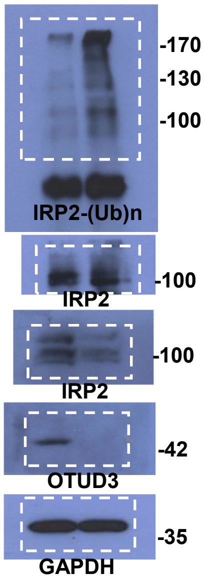

Fig 4d.

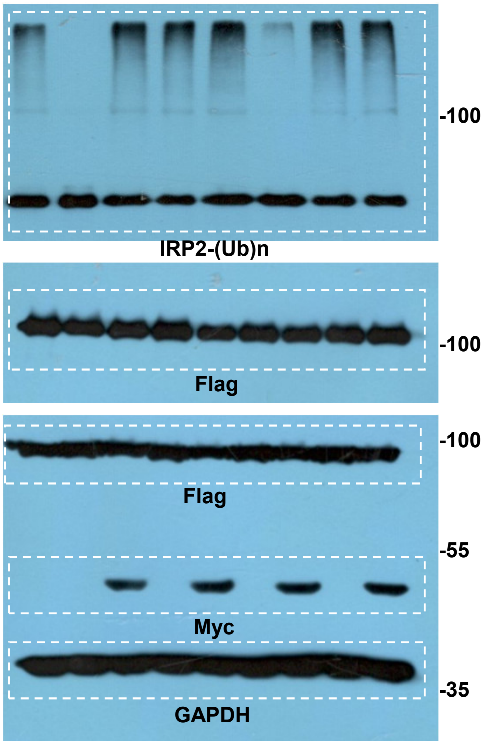

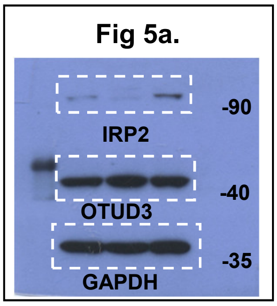
Fig 5a.
-90
IRP2
-40
OTUD3
-35
GAPDH

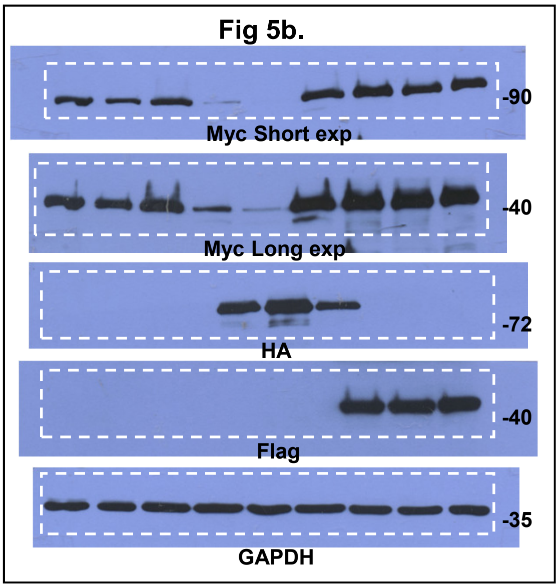
Fig 5b.
-90
Myc Short exp
-40
Myc Long exp
-72
HA
-40
Flag
-35
GAPDH

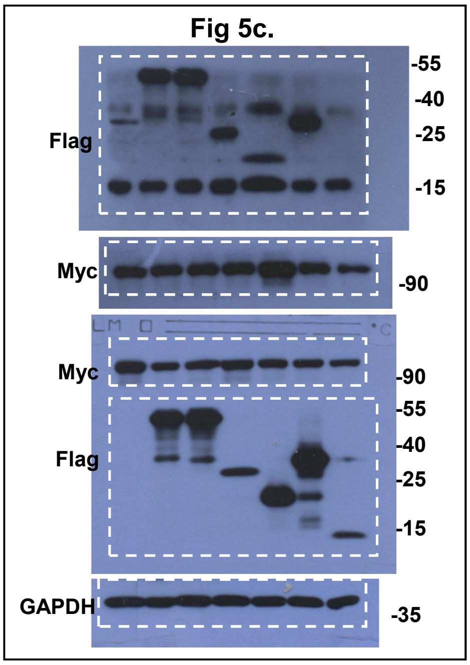
Fig 5c.
-55
-40
-25
-15
Flag
Myc
-90
Myc
-90
-55
-40
-25
-15
Flag
GAPDH
-35

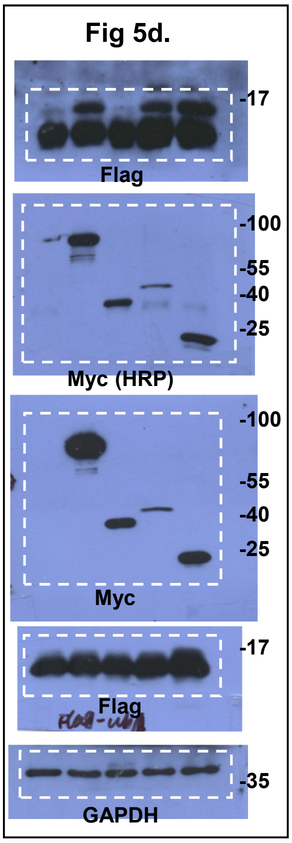
Fig 5d.
-17
Flag
-100
-55
-40
-25
Myc (HRP)
-100
-55
-40
-25
Myc
-17
Flag
-35
GAPDH

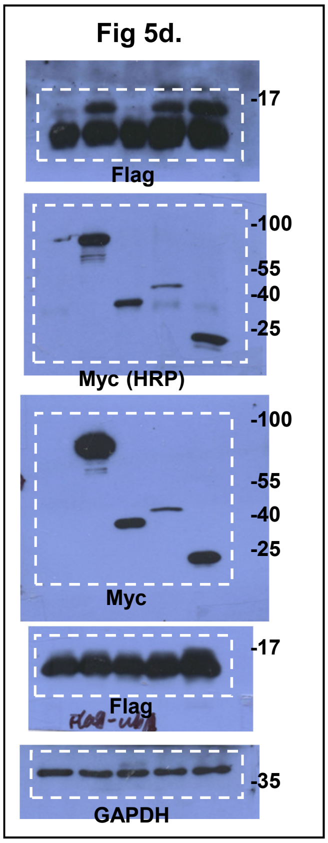
Fig 5d.
-17
Flag
-100
-55
-40
-25
Myc (HRP)
-100
-55
-40
-25
Myc
-17
Flag
-35
GAPDH

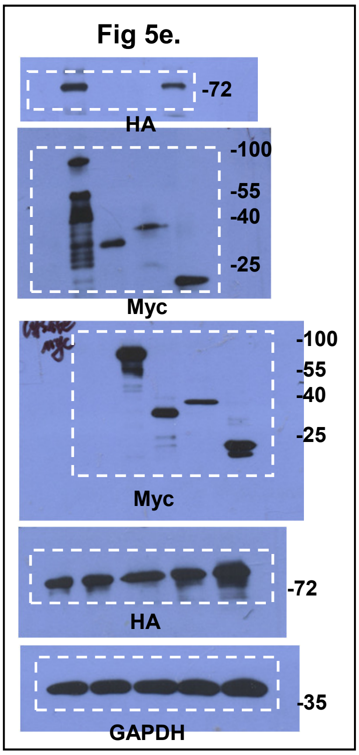
Fig 5e.
-72
HA
-100
-55
-40
-25
Myc
-100
-55
-40
-25
Myc
-72
HA
-35
GAPDH

Supplementary Fig 1f.

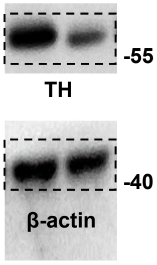

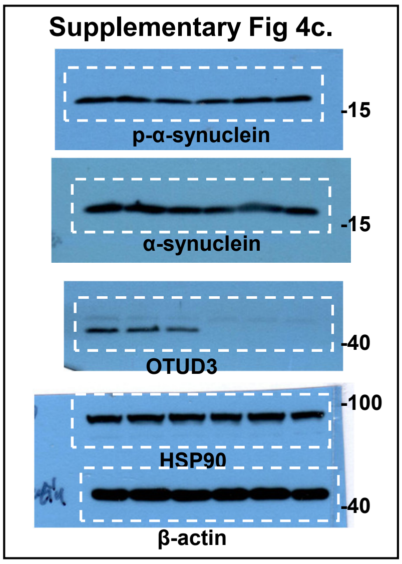
Supplementary Fig 4c.
-15
p-α-synuclein
-15
α-synuclein
-40
OTUD3
-100
HSP90
-40
β-actin

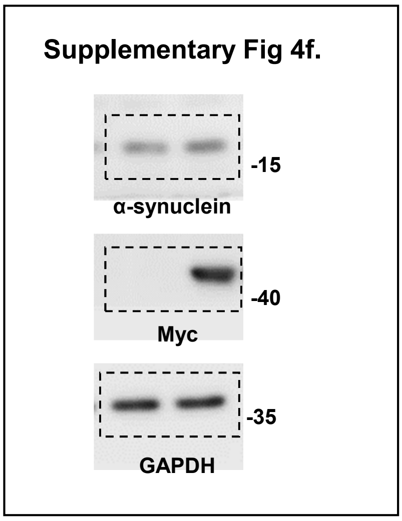
Supplementary Fig 4f.
-15
α-synuclein
-40
Myc
-35
GAPDH

Supplementary Fig 5f.

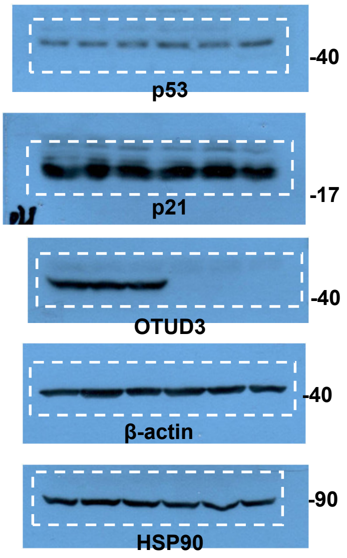

Supplementary Figure 5h.

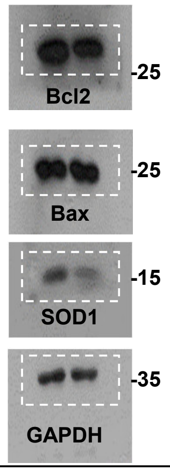

Supplementary Fig 5i.

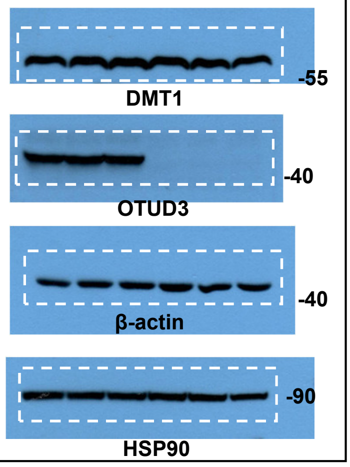

Supplementary Fig 5k.

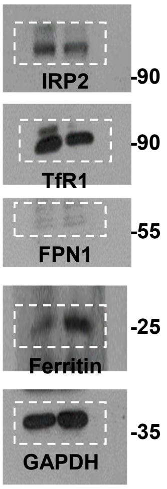

Supplementary Fig 5l.

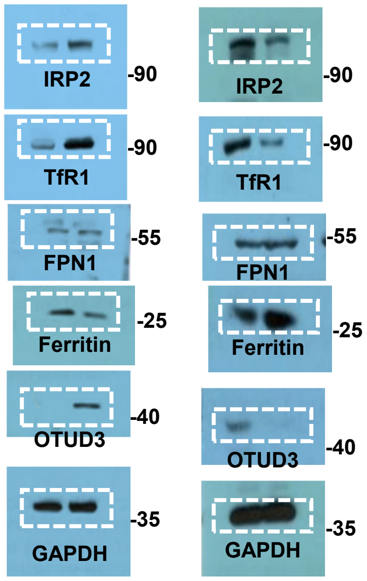

Supplementary Fig 6a.

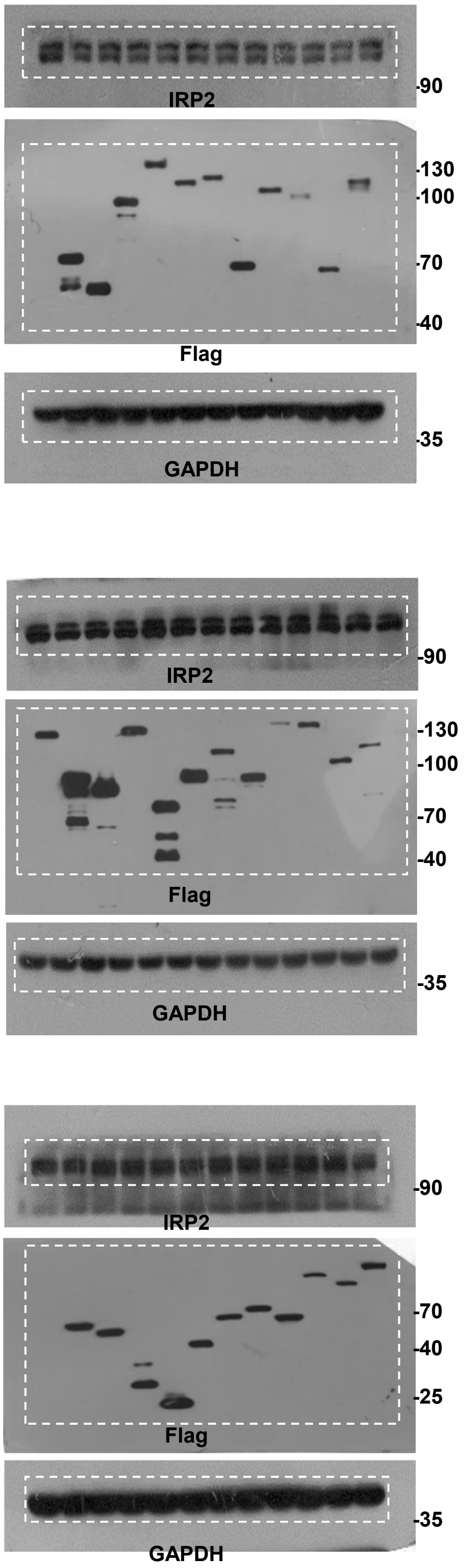

Supplementary Fig 6b.

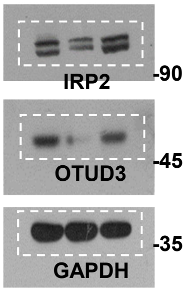

Supplementary Fig 6e.

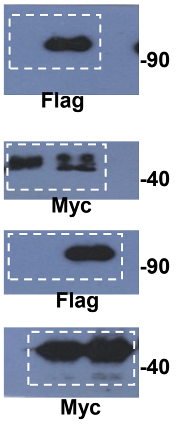

Supplementary Fig 6f.

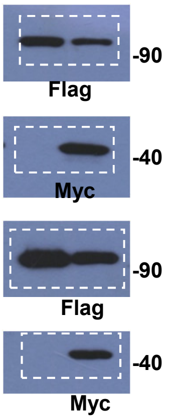

Supplementary Fig 7a.

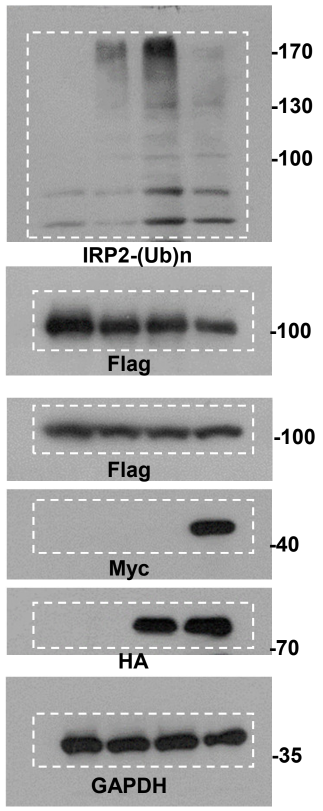

Supplementary Fig 7b.

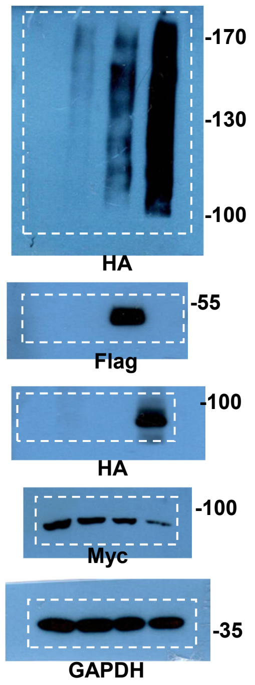

Supplementary Fig 7c.

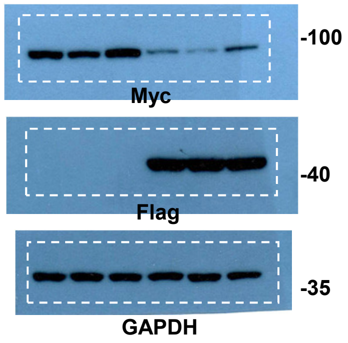

Supplementary Fig 7d.

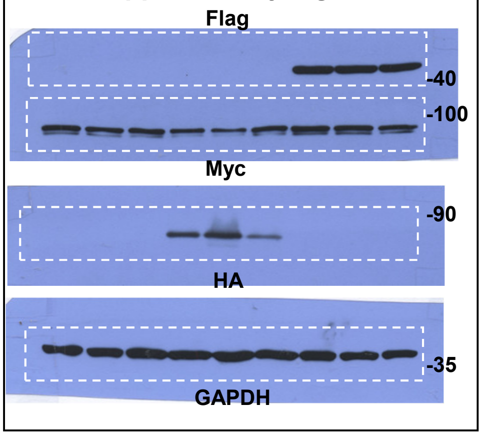

Supplement: Supplementary file 6 — Uncropped Western Blots [file 41419_2022_4704_MOESM6_ESM.pdf]
